# Current Understanding of the Molecular Basis of Venezuelan Equine Encephalitis Virus Pathogenesis and Vaccine Development

**DOI:** 10.3390/v11020164

**Published:** 2019-02-18

**Authors:** Anuj Sharma, Barbara Knollmann-Ritschel

**Affiliations:** Department of Pathology, Uniformed Services University of the Health Sciences, Bethesda, MD 20814, USA; barbara.knollmann-ritschel@usuhs.edu

**Keywords:** Venezuelan equine encephalitis virus, alphavirus, inflammation, encephalitis, viral and host factors, blood brain barrier, pathogenesis, vaccines

## Abstract

**Dedication:**

This review is dedicated in the memory of Dr Radha K. Maheshwari, a great mentor and colleague, whose passion for research and student training has left a lasting effect on this manuscript and many other works.

**Abstract:**

Venezuelan equine encephalitis virus (VEEV) is an alphavirus in the family Togaviridae. VEEV is highly infectious in aerosol form and a known bio-warfare agent that can cause severe encephalitis in humans. Periodic outbreaks of VEEV occur predominantly in Central and South America. Increased interest in VEEV has resulted in a more thorough understanding of the pathogenesis of this disease. Inflammation plays a paradoxical role of antiviral response as well as development of lethal encephalitis through an interplay between the host and viral factors that dictate virus replication. VEEV has efficient replication machinery that adapts to overcome deleterious mutations in the viral genome or improve interactions with host factors. In the last few decades there has been ongoing development of various VEEV vaccine candidates addressing the shortcomings of the current investigational new drugs or approved vaccines. We review the current understanding of the molecular basis of VEEV pathogenesis and discuss various types of vaccine candidates.

## 1. Introduction

**Figure 1:**
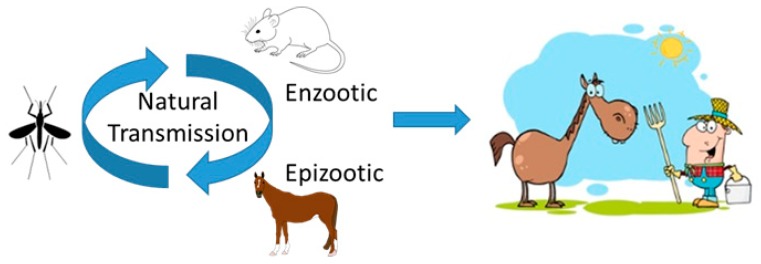
Venezuelan equine encephalitis virus (VEEV) transmission. VEEV is transmitted by a variety of mosquitoes. Enzootic strains are maintained in a cycle between small rodents and mosquitoes. Epizootic strains are transmitted by mosquitoes to equines, causing high titer viremia and high mortality. The virus is tangentially transmitted by mosquitoes from equines to humans that work in close contact with equines.

**Figure 2:**
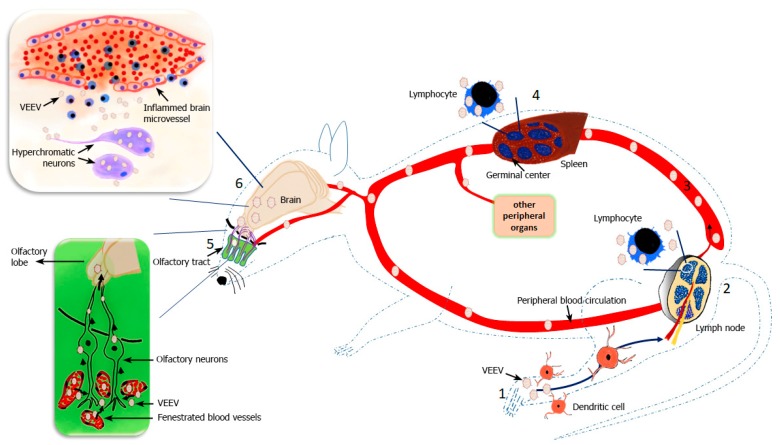
Biphasic replication of VEEV in mice. Mice infected via VEEV footpad injection mimics the natural mode of transmission by mosquito bites (1). After initial inoculation, regional dendritic cells take up the virus and transport it to the local draining lymph nodes, where VEEV replicates in lymphocytes (2) and is then released into the circulation resulting in viremia (3). VEEV replicates in various tissues, but lymphoid organs such as the spleen are primary replication sites (4). Around 24–36 h post infection, VEEV escapes into the olfactory tract infecting olfactory nerve endings initiating the first step of CNS phase of infection (5). Virus moves through the axons of the olfactory neurons and enters the olfactory lobe of the brain (large insert). In the brain, VEEV primarily infects the neurons, but glia and oligodendrocytes are also targets of VEEV infection. VEEV induced inflammation causes vascular cuffing and alteration in the BBB allowing mononuclear lymphocytes to enter the brain (6). VEEV mediated alteration of the BBB may allow the virus to enter the brain, but this route of entry is debated.

Venezuelan equine encephalitis virus (VEEV) is a member of genus *Alphavirus* in the family Togaviridae. VEEV complex is a group of 14 antigenic varieties divided into 7 species. The VEEV species include four antigenic varieties namely IA/B, IC, ID, and IE, all of which cause human disease that is indistinguishable between the antigenic varieties [1]. Subtypes IA/B and C are epizootic strains that cause fulminant disease and high mortality in equines. Subtypes ID and IE are enzootic strains that are typically avirulent in equines; however, IE can be neurovirulent in equines. VEEV is an enveloped virus which is maintained in nature in a cycle between rodents and mosquitoes with epizootic strains sporadically causing outbreaks in equines and humans (Figure 1) [2,3]. The geographic distribution and outbreaks of VEEV in equines and humans has been reviewed in detail by Aguilar et al. [1] and Weaver et al. [4]. VEEV is a Category B agent as defined by the Centers for Disease Control and Prevention, and National Institutes of Health. Biosafety level 3 containment is required for handling of live virulent strains of VEEV. Two live-attenuated strains of VEEV, namely TC-83 and V3526, can be safely handled at biosafety level 2 containment [5]. VEEV infection in humans starts with an asymptomatic incubation period of 1–5 days followed by the onset of a febrile illness characterized by fever, headache, nausea, vomiting, myalgia, ocular pain, lower back pain and diarrhea lasting for 1–4 days [6]. The short febrile illness may progress into fulminant encephalitis causing convulsions, hemiparesis, behavioral changes, and alteration of consciousness. A more severe infection can occur which is associated with hemichorea, seizures, and stupor or coma [7,8,9]. Mortality in humans is <1%, but the incidence of neurological disease can be up to 14% in infected patients [10]. The mouse is the most common model used to investigate VEEV pathogenesis as it closely mimics the biphasic course of peripheral replication followed by infection of the central nervous system (CNS) as seen in severe cases of human VEEV infection i.e., the initial febrile illness due to virus replication in the peripheral organs followed by a second phase of CNS infection (Figure 2) [11]. In healthy immunocompetent adult mice models such as CD-1 Swiss [12], Balb/c [13], and C57BL6 [14] mice, infection with wild-type VEEV causes a biphasic disease similar to the severe form of disease in humans. VEEV can be detected in local lymph nodes as early as 6 h post infection. Animals become viremic within 12 h of infection. By 12 h post infection, VEEV can also be detected in other peripheral organs. The virus replicates in the lymphoid tissue e.g., lymph nodes and spleen, as well as in non-lymphoid organs including the heart, lung, kidney, and pancreas. In the lymphoid tissues, VEEV induces cellular necrosis and an inflammatory cell response. Loss or alteration of germinal center structures in the spleen is observed as early as 24 h post infection and is accompanied by lymphocyte karryohrexis and apoptosis, as well as macrophage infiltration. Recovery starts by 72 h post infection. The virus is cleared from peripheral organs within 4–5 days of infection. In the brain, VEEV first appears in the olfactory lobe around 36–48 h post infection. The virus then spreads rapidly throughout the brain. Perivascular cuffing and lymphocyte infiltration are observed 72 h post infection. Viral spread and corresponding inflammation are characterized by perivascular lymphocytic cuffing, gliosis, neurodegeneration, and vacuolization of neuropil, which increase in intensity with time. The kinetics of viral spread into the brain is dependent on the route of infection. Virus appears in the CNS much earlier when infection is via aerosol exposure due to the direct infection of olfactory neuroepithelium by aerosolized of VEEV particles, compared to a subcutaneous infection route which requires virus replication in lymphoid tissue and the development of viremia in order for the virus to then be able to infect the olfactory neuroepithelium [13,15,16]. Additionally, extensive hepatocellular degeneration and interstitial pneumonia are seen in human VEEV infections; however, these are not the primary pathological findings in animal models used for investigating VEEV infection [17]. Characterization of VEEV pathogenesis has been aided by the availability of clearly defined mutant strains developed by point mutations in the full-length clone of wild-type Trinidad Donkey (TrD) strain (subtype I/AB) of VEEV (Table 1), which based on the extent of attenuation shows differential replication kinetics in mice ranging from a mild febrile illness due to limited peripheral replication to a full blown encephalitis [18,19,20]. This review will focus on the advances made in understanding the molecular basis of VEEV pathogenesis and discussion of recent vaccine candidates.

## 2. Innate Immune Response to VEEV Infection and the Role of Interferon

The innate immune response is an important first line of defense against pathogens in the form of cytokine release and ensuing inflammation which can either be protective of the host by killing virus-infected cells and producing an antiviral state mediated by interferon release, or it can be detrimental to the host causing tissue damage by cytokine storm. These paradoxical roles of immune response are evident during VEEV infection. Natural Killer (NK) cells are an important component of the innate immune response and are responsible for killing virus-infected cells by direct recognition [24]. Protective NK-cell activity has been suggested to occur following VEEV infection. NK-cells activated by the cell-free supernatant of splenocytes from mice immunized with inactivated TC-83, a live-attenuated strain of VEEV, protected syngeneic and allogeneic mice as well as nude mice from a lethal virulent TrD strain challenge [25]. Treatment with the immunomodulatory compound 3,6-bis (2-piperidinoethoxy) acridine trihydrochloride, which is a strong activator of NK-cells, protected mice against a lethal intra-peritoneal challenge with VEEV [26]. Natural killer T (NKT)-cells are cytotoxic cells that express cell surface markers for both NK and T-cells. Infection of NKT-cells with VEEV replicon particles activates a cytokine response that is more similar to NK-cells than T-cells, further suggesting there is activation of NK-cells by VEEV [27]. In contrast to these studies, NK-cells have been suggested to increase the severity of VEEV infection in the CNS of C3H/HeN mice [28]. C3H/HeN mice are a cross of Dilute Brown Non-Agouti (DBA) and Bagg albino mice, and are homozygous for the retinal degeneration 1 (*Pde6b^rd1^*) gene mutation, which causes spontaneous retinal degeneration in these mice. Infection of C3H/HeN mice with TC-83 via aerosol and intranasal routes is lethal and the cytokine profile in the brain tissue suggests NK-cell activity [29,30]. Depletion of NK-cells in these mice improved the survivability after intranasal TC-83 infection, suggesting a pathogenicity-enhancing role of NK-cells in the CNS. However, TC-83 is non-lethal in C3H/HeN mice when injected subcutaneously. It is possible that due to intranasal inoculation, TC-83 can evade the antiviral activity of the NK-cells which it would otherwise encounter during peripheral replication. During the CNS phase of infection, the activated NK-cells may contribute to neuroinflammation by releasing proinflammatory cytokines. It is known that the inflammation contributes to the severity of the VEEV infection in the brain, and increased inflammation due to the influx of activated NK-cells could explain the enhanced encephalitis resulting in death of C3H/HeN mice following intranasal infection with TC-83 [28,31,32].

The complement system is another important arm of the innate immune response for identifying and eliminating pathogens. Activated complement aids in opsonization and subsequent lysis of pathogens, eliciting an inflammatory cytokine response, immune surveillance and homeostasis [33,34]. Several viruses exhibit molecular mimicry such that the viral proteins have structural and functional homology to the host complement regulatory proteins, resulting in the evasion of detection by the complement system [35]. The protective role of complement C3 in VEEV infection was demonstrated using an attenuated VEEV strain: V3533. V3533 was isolated as a revertant mutant of the V3010 strain of VEEV, containing the envelope (E) 2 Lys 76 mutation of V3010, and an additional Lys to Glu mutation at the E2 116 position. Infection of immunocompetent CD-1 mice with V3533 caused moderate mortality and the virus was isolated from the lymphoid and brain tissue [20]. In the absence of complement C3, subcutaneous injection of V3533 resulted in high viremia titers, an early appearance in the brain and spinal cord, and prolonged viremia compared to infection in the wild-type mouse with functional C3. High virus titers were also associated with more severe symptoms of the disease; however, animals eventually cleared the virus and recovered. Interestingly, intracranial injection of C3^-/-^ mice with V3533 does not affect virus titers or disease severity from similarly infected wild-type mice, suggesting that the antiviral property of C3 is limited to early virus replication in the peripheral organs, and once the virus has entered the brain, complement has no effect on VEEV replication [36].

Interferon (IFN) is an innate antiviral cytokine that is protective against VEEV infection. VEEV infection has been shown to induce both type I and II IFN, and several IFN regulatory factors (IRF) are modulated during VEEV infection [37,38,39,40,41]. Mice lacking the type I IFN receptor, or functional IRFs, are highly sensitive to VEEV infection and show rapid spread, enhanced pathology, failure to clear the virus from circulation, and a shortened mean survival time following infection with virulent, as well as avirulent, strains of VEEV. However, the protective effect of IFN is observed only with the endogenous production of IFN as mice treated with exogenous IFN do not show increased protection or survival, except in C3H/HeN mice. In C3H/HeN mice treatment with IFN-α induces a dose-dependent protection against intranasal infection with TC-83, and this could be because of the low pathogenic potential of TC-83 compared to the virulent wild-type VEEV [29,42,43]. Although endogenous IFN is produced in response to VEEV infection, VEEV inhibits IFN signaling by inhibiting signal transducer and activator of transcription 1 (STAT1) and Janus kinase (Jak)1/2 phosphorylation, and nuclear localization of STAT1 [44]. This may explain the ineffectiveness of exogenous IFN in treating VEEV infection. In vitro, induction of IFN by VEEV depends on and varies with types of cells [42,45]. VEEV inhibits IFN expression in human primary neuronal cells and although, pretreatment of these cells with IFN inhibited VEEV replication, the extent of inhibition was marginal compared to the Sindbis virus (SINV) replication. However, no antiviral effect was observed when these cells were treated with IFN after infection, which correlates with the inhibition of phosphorylation of STAT1/2 and reduced expression of interferon stimulated genes (ISG). The differential IFN induction and resistant to an antiviral state induced by pretreatment with IFN may explain a greater neurovirulence of VEEV among alphaviruses [45].

In summary, it is clear that the innate immune responses play an important role in protection from VEEV infection during early virus replication in peripheral tissues. It is however, not fully understood how wild-type infectious VEEV evades or overcomes these early innate antiviral responses and replicates to high enough titers in the serum that enables the virus to cross into the olfactory tract and eventually into the brain. Early replication of the virus in the brain is protected from the peripheral immune response due to the immune privileged state of the CNS, enabling the virus to replicate in its target cells i.e., the neurons and glia. With progression of infection, there is opening of the blood brain barrier (BBB) allowing influx of activated peripheral lymphocytes. However, in infections with virulent VEEV strains, influx of peripheral immune cells appears to have very little effect on restricting viral replication and axon-to-axon neuronal spread in the brain. Rather, the influx of peripheral immune cells contributes more to increasing the inflammation in the brain, further augmenting the encephalitis.

## 3. Role of Alphavirus Genes in Infection

**Figure 3:**
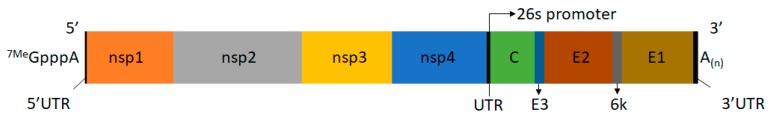
**Organization of VEEV genome**. VEEV genome is a single-stranded positive-sense RNA of 11.4kb length. VEEV encodes four nonstructural proteins (nsP1, nsP2, nsP3 and nsP4) and five structural proteins (capsid (C), envelope (E) 3, E2, 6k, and E1). VEEV genome has mRNA characteristics. 5’ untranslated region (UTR) is capped with methyl residue present on the 7-position of capping guanosine nucleotide. A short UTR is present after the nsp4 gene sequence that has a promoter sequence for a 26S subgenomic RNA. There are two open reading frames (ORF) in the genome. The first ORF in the 5’ region translate nonstructural proteins and the second ORF in sub genomic RNA translate structural proteins. 3’ end of the genome has a poly(A) tail.

**Figure 4:**
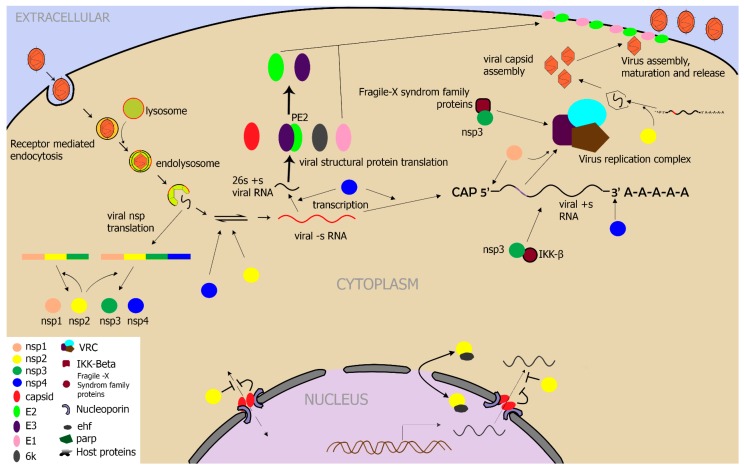
**Replication of VEEV in host cell**. VEEV enters in the host cells by receptor mediated binding of the virus to the host cell membrane. Virus containing endosomes fuse with lysosomes resulting in formation of endolysosomes. Viral RNA is released in the cytoplasm following pH dependent conformational changes in the viral proteins, allowing fusion with the endolysosome membrane. VEEV is a single-stranded positive-sense RNA virus that replicates in the cytoplasm and does not have a nuclear phase of replication. In the second phase of infection, viral nsp are translated as P123 and P1234 polyproteins from the viral genomic RNA. Autolytic activity of nsp-2 cleaves the viral polyproteins in individual nsp-1, nsp-2, nsp-3, and nsp-4 proteins. Nsp-4 is a viral RdRp, which with methyltransferase activity of nsp-2, drives synthesis of negative-sense viral RNA. Negative-sense viral RNA is transcribed into smaller 26S subgenomic positive-sense RNA and a full-length positive-sense viral RNA. Subgenomic 26S RNA is translated into viral structural proteins namely capsid, a polyprotein of E3 and E2 called PE2, 6k, and E1. PE2 is processed into E3 and E2 proteins by cutting at the furin-cleavage site in PE2. Viral nsp-2 plays a role in capping of viral genomic RNA via its methyltransferase and guanylylation activity. Nsp-4 adds a poly-A tail to the viral genomic RNA via its terminal adenyltransferase activity. Full length positive- sense viral RNA is incorporated into a virus replication complex (VRC). Assembly of VRC is aided by interaction of viral non-structural proteins with host proteins such as nsp-3 with IKK-β and Fragile- X syndrome family proteins. Nsp-3 binds to other unknown host proteins during formation of VRC, the role of which is not yet understood. Nsp-1 conserved sequence element helps in the recognition of the core promoter element of the virus genome by VRC. In addition to direct involvement of viral proteins in replication and assembly, viral non-structural proteins interact with host factors to promote VEEV replication. Capsid proteins bind to components of the nuclear pore complex effectively blocking nuclear-cytoplasm-nuclear traffic and host protein translation. Nsp-2 interacts with karyopherin-alpha 1 for its nuclear localization function, role of which is not clearly understood. Nsp2 plays a role in loading of the viral RNA into nucleocapsid and maturation of virions. In the final step structural proteins E1 and E2 are embedded in the plasma membrane, and assembly and release of the mature virion particle occurs by encapsulating nucleocapsid and budding at plasma membrane [2].

VEEV is a single-stranded positive-sense ribonucleic acid (RNA) virus with four nonstructural genes and five structural genes (Figure 3) [46,47]. Early events of alphavirus infection (within hours), are important in establishing the infection. Outcome of the viral infection is dependent on an intricate interaction of antiviral and proviral responses which, can either create an antiviral state or help the virus to replicate, respectively (Figure 4). Viruses have evolved mechanisms to overcome the host antiviral responses thus establishing productive virus replication [48]. Alphaviruses negatively regulate the host cytoplasm-to-nuclear transport, and vice versa, which prevents (a) signal transduction into the nucleus suppressing the transcription of host genes, and (b) transport of cellular mRNA into the cytoplasm suppressing translation of host genes [49,50]. VEEV structural proteins inhibit transcription, and in the absence of capsid, nonstructural proteins (nsp) inhibit translation. The extent of host macromolecular synthesis shutoff, however, varies with cell type [45]. The host gene transcription and protein translational shutoff is mediated by the capsid protein of VEEV [50,51]. One of the most prominent phenotypic observations in VEEV-infected cells is the development of cytopathic effects evident by the dislodging, rounding off and lysis of infected cells. The capsid protein carries a 39 amino acid long sequence termed H68, which contains a nuclear localization signal (NLS) and a nuclear export signal (NES) that are responsible for interacting with the host cell nuclear import and export proteins [49,52,53]. VEEV capsid protein binds to importin α/β and exportin-1 (also known as chromosomal maintenance-1 protein), and nucleoporins of the nuclear pore complex, resulting in blockage of nuclear-to-cytoplasmic transport [49,50,54]. This causes host translational shutoff inducing cytopathic effect in the infected cells. Mutation in the NLS and connecting peptide between the NLS and NES, or only in NES changes the phenotype of TC-83 strain of VEEV from lytic to persistent non-cytopathic infection in mammalian cells suggesting importance of these domains in lytic phenotype of the virus [55]. Targeted inhibition of host nuclear export proteins interfered with the cellular localization of the capsid and decreased replication of VEEV and other New World alphaviruses. However, this inhibition was overcome by adaptive mutations in the nuclear localization and export signals of the capsid protein [54]. In addition, VEEV has evolved to be resistant to the antiviral state induced by pretreatment of cells with IFN [45]. VEEV nonstructural protein (nsp) 2 is important for the resistance that VEEV shows towards IFN-induced antiviral state, and mutation in the *nsp2* gene reinstates the sensitivity to IFN-induced antiviral state in cells. Nsp2 alone also induces a host macromolecular synthesis shutoff, similar to that of the capsid protein [56]. Therefore, it appears that both nsp2 and capsid proteins of VEEV inhibit host cell translation and may synergistically act to shutoff the host macromolecular synthesis activity.

Alphaviruses, including VEEV, use programmed -1 ribosomal frameshift (-1 PRF) for encoding the viral *trans*-frame (TF) protein. TF protein is made by the presence of a slippery codon motif, UUUUUUUA, in the *6K* gene, which mediates a ribosomal frameshift into -1 open reading frame (ORF). TF, therefore, is 6K protein with 15 additional amino acid residues at the C-terminus. The slippery codon motif in the *6K* gene and a stop codon in the -1 ORF sequence of *E1* gene are highly conserved among alphaviruses [57]. Mutations in PRF of the TrD strain of VEEV reduces virus titers in vitro probably secondary to a defective virus assembly, as no effect on viral RNA replication is noted. In contrast, mutations in PRF of TC-83 strain does not show such an effect on virus replication, which could be due to compensation by the presence of additional mutations in the envelope proteins of TC-83. PRF mutated TrD strains show attenuated infectivity in mice, as well as a significant reduction in mortality, which is accompanied by a reduced amount of virus in the blood, spleen and brain of infected mice [58]. It is not clear if the TF protein itself plays a role in the virus replication, or if it regulates levels of envelope protein-1 (E1), nevertheless -1 PRF seems to be an important aspect of VEEV pathogenicity especially in the wild-type strains of VEEV.

Among alphaviruses, VEEV has very efficient virion formation. Virion assembly is an important step in virus replication and depends on multiple factors including efficacy of virus replication complex, assembly and release [59]. VEEV nsp1-3 are important for packaging viral RNA into a nucleocapsid complex, therefore, have important roles in virus replication. Using an array of defective helper RNA systems, Volkova et al. demonstrated that the presence of *nsp1-3* in *cis* formation increases the efficiency of replication of VEEV RNA when *nsp4*, which encodes for viral RNA dependent RNA polymerase (RdRp), is provided in *trans* [60]. The *Nsp1* gene of VEEV contains a 51-nt conserved sequence element (CSE) that is essential for virus replication. Mutation or deletion of CSE results in loss of infectivity of VEEV, probably due to inefficient recognition of the viral RNA core promoter element by the virus replication complex. However, an adaptive mutation in the *nsp2* gene sequence, and to some extent in *nsp3*, can overcome deleterious mutations in the 5’ untranslated region (UTR) and 51-nt CSE of *nsp1* [61,62]. Nsp1 functions as a methyltransferase and guanylylation enzyme, two properties needed for the 5’ capping of VEEV genomic RNA [63].

The nsp2 protein of alphaviruses has multiple important functions. Nsp2 has protease activity that processes the nsp1234 and nsp123 polyproteins into individual nsp1, nsp2, nsp3 and nsp4 proteins [64,65]. Nsp2 has methyltransferase activity and is responsible for regulation of the minus strand synthesis [66]. During VEEV infection, nsp2 shuttles between the cytoplasmic and nuclear compartments of the infected cells. While the exact function of this process is not known, it is suggested to either aid in virus replication by recruiting essential host factors needed for viral replication, or inhibit transcription of host antiviral response genes, thus helping in evasion of the cellular antiviral response [67]. Additionally, nsp2 is involved in the maturation of VEEV-virions by regulating packaging of the viral genomic RNA into virion particles [68].

The *nsp3* gene of VEEV contains a hyper variable domain (HVD), which is unique to VEEV and shows little homology with other alphaviruses. The C-terminus domain of the HVD is essential for VEEV replication [69]. The HVD shows selective influence on VEEV’s capability to replicate in cells, such that in highly permissible vertebrate cell lines, the presence of HVD is insignificant and can be deleted without deleterious effect on virus replication. In mosquito cells however, modification of the HVD negatively affects virus replication. Although such deleterious modifications in the HVD have been shown to overcome by adaptive mutations elsewhere in the gene, resulting in competent virus replication. Additionally, HVD determines interaction of nsp3 protein with cellular proteins during formation of the cytoplasmic complexes that are needed for virus replication [70].

Not much has been specifically studied about VEEV nsp4. Analogous studies in SINV and Semliki forest virus (SFV) show that nsp4 is an RdRp responsible for the synthesis of plus and minus strands of viral RNA. Nsp4 has terminal adenyltransferase activity adding a polyA tail to the viral RNA genome. Nsp4 may also have an auto proteinase activity responsible for cleaving nsp3-nsp4 polyprotein into individual proteins [71,72,73,74].

## 4. Role of Cellular Factors in VEEV Replication

Viruses need host cellular proteins/factors for successful infection and replication. This is more evident for RNA viruses that are entirely dependent on host cellular translation machinery for replication. Interaction of viral proteins or genome with cellular factors determines almost every step of the virus infection and replication cycle, starting from the attachment of the virus to cellular surface, to the release of mature virions from infected cells. VEEV uses laminin binding protein as the putative receptor for infecting both mosquito and mammalian cells [75,76]. VEEV entry is pH dependent, using clathrin-mediated endocytosis, and requiring early and late endosome formation for its entry into the host cell. In addition, VEEV entry is cholesterol-independent and viral RNA is released into the cytoplasm by low cholesterol containing late endosomes [77]. Binding to heparin sulfate (HS) has been described for adaptation of VEEV in tissue culture. Tissue culture-adapted low-virulence strains of VEEV show a high affinity for binding with HS, a property that presumably aids in rapid clearance of tissue culture-adapted strains from the host [78]. High affinity binding of tissue culture-adapted VEEV with HS is common. Similar observations have been made in other alphaviruses where repeated passages and adaptation to tissue culture results in positive charge substitution mutations in E2 glycoprotein of the virus resulting in increased binding with HS during infection [79]. However, not all alphaviruses show attenuation associated with increased HS interaction. Virulence of naturally occurring Eastern equine encephalitis virus (EEEV) is dependent on binding with HS, and infectivity of SINV in mosquitoes and mice is dependent on the HS binding affinity of viral glycoproteins [80,81,82]. Nevertheless, the increased binding capacity of viral glycoproteins to HS is always observed during the adaptation of alphaviruses to tissue culture. Other cellular factors influence VEEV infection as well. Nuclear factor kappa B (NfkB) activation has been shown in response to VEEV infection [37]. Activation of NfkB is regulated by IkB kinase (IKK) [83]. A study by Amaya et al. showed that a component of the IKK complex, called IKK-β, involved in the phosphorylation of the p65 subunit of the NfkB transcription complex, interacted with the nsp3 protein of VEEV during infection. Treatment of glioblastoma and neuronal cells with IKK-β inhibitors inhibited replication of TC-83 and TrD strains of VEEV. Replication of TC-83 strain of VEEV in C3H/HeN mice was also reduced by IKK-β inhibitor resulting in increased survival after infection [84]. Fragile X mental retardation (FMR) and fragile X-related (FXR) proteins are RNA binding proteins that participate in formation of ribonucleoprotein complex (RNP) [85]. Members of Fragile X syndrome family proteins (FXR1, FXR2, and FMR1) co-localize with VEEV nsp3 complexes in infected cells, and this function is redundant between the FXR family proteins. FXRs help in the assembly of virus replication complex (VRC) by binding with the viral RNA. This function was found to be dependent on two short repeat sequences in the C-terminus of VEEV *nsp3* gene [86]. Host cellular DEAD-box RNA helicase (DDX) -1 and -3 have also been implicated in VEEV replication. DDX-1 and -3 immunoprecipitated with the TC-83 nsp3 protein, and depletion of DDX-1 and -3 using short interfering RNA (siRNA) reduced TC-83 replication. A similar experiment with TrD strain of VEEV also showed reduction in virus replication, albeit reduction in TrD replication due to DDX-3 depletion was not as strong as observed for TC-83. Effect of double depletion of DDX-1 and -3 against TC-83 replication was likewise more significant than that observed for TrD strain of VEEV. Co-precipitation experiments elucidated the presence of nsp3 of TC-83 in complex with host translation machinery, including DDX-3, suggesting association of nsp3-DDX-3 with host translation machinery for the translation of viral proteins [87]. A host protein, poly (ADP-ribose) polymerase (PARP) 12 (PARP-12) has been shown to negatively regulate VEEV replication. Expression of PARP-12 and bone marrow stromal antigen 2 (Bst2, also known as tetherin) genes was found to be elevated in cells infected with a clone of TC-83 that replicates without causing cytopathic effects. When PARP12 was expressed either as a cloned gene within TC-83 genome or via a separate expression plasmid, replication of the virus was significantly reduced. Other members of PARP family viz., PARP-7 and 10, also showed similar anti-VEEV activity. This antiviral effect of PARP was suggested to be due to suppression of cellular translation via complex formation with polysomes and RNA molecules [88,89]. However, in these studies the antiviral effect of PARP overexpression on wild-type VEEV was not shown, and it remains to be seen if PARP overexpression may reduce the replication efficiency of the wild-type VEEV. IFN-induced transmembrane 3 (*Ifitm3*) gene is an ISG, which is expressed in the brains of mice infected with V3000 strain of VEEV [16]. Replication of the TC-83 strain of VEEV is enhanced in *ifitm3-del* or *ifitm3^-/-^* mouse embryonic fibroblast cells treated with IFNβ compared to wild-type fibroblast cells. The moderately pathogenic mutant of TC-83 strain of VEEV (VEEV-TC-83-A3G) caused higher morbidity and mortality in *ifitm3^-/-^* mice compared to the wild-type mice. Increased morbidity and mortality were associated with high titers of VEEV-TC-83-A3G in various organs of the *ifitm3^-/-^* mice [90]. In a comparative study of neurovirulent vs. partially neurovirulent strains of VEEV, *ifitm3* expression was observed only in the brains of mice infected with the neurovirulent V3000 strain of VEEV [16]. It is possible that the partially neurovirulent VEEV strain did not replicate to high enough titers in these brains to stimulate measurable levels of *ifitm3*. An interesting observation was reported in a VEEV pathogenesis study in macaques that higher levels of testosterone prior to VEEV infection correlated with higher levels of viremia following exposure to aerosolized wild-type TrD strain of VEEV. In addition, the authors noted that the levels of testosterone fell following exposure to the virus [91]. The authors suggested that high levels of testosterone in males may induce a transiently increased susceptibility to VEEV infection; however, detailed studies are needed to make such conclusions. Collectively, these studies show an interplay of VEEV proteins, especially nsp3, with various host proteins that positively affect the virus replication. Although inhibition of such interactions reduces virus replication (attenuated and/or wild-type), a total loss of infectivity has not been observed, and in many instances a considerable viral titer is still produced in cell supernatants. This suggests that there are multiple redundant replication mechanisms that allow for the virus replication to occur in the absence of a preferred mode of replication. The cellular proteins such as DDX1/3, FXRs, and IKK-β, therefore provide viable targets for developing host-specific anti-VEEV therapeutic drugs which can aid in recovering from VEEV infection by slowing down or reducing virus replication in the event of an outbreak or deliberate dispersion of VEEV.

## 5. CNS Infection of VEEV and the BBB

Entry of VEEV into the CNS has been studied in detail using neurovirulent TrD strain (subtype I A/B). VEEV primarily uses the olfactory system to enter the CNS irrespective of the route of infection. It is believed that the fenestrated epithelial lining of the blood vessels at this site provides access for VEEV to infect the neurons of the olfactory neuroepithelium [12,92]. In the later stages of infection, or in the absence of olfactory neurons, VEEV uses the trigeminal nerve to enter CNS [12]. Both these routes are not unique to VEEV as several other viruses such as influenza, herpes, polio, vesicular stomatitis, rabies, and Japanese encephalitis viruses use olfactory neurons or the trigeminal nerve to enter CNS [93,94,95,96,97,98]. VEEV entry into the brain via BBB is debated. An early study by Gorelkin using electron microscopy showed that the wild-type neurovirulent TrD strain of VEEV infected the endothelial cells of the BBB. The appearance of VEEV in endothelial cells was concomitant with the appearance of perivascular cuffing and edema, suggestive of early BBB alteration [99]. Ryzhikov’s group later proposed that an extremely low-level penetration of VEEV thorough BBB occurred after aerosol exposure with the TrD strain of VEEV [92]. Experiments with replication-deficient VEEV replicon particles showed that VEEV infection-induced breakdown of the BBB could result in the invasion of the brain by virus particles that are circulating in the blood [100]. Other studies, however, downplayed the role of the BBB as a route of VEEV entry into the brain due to the lack of localization of viral antigen in the endothelial cells of the BBB [30,101]. Nevertheless, the BBB plays an essential role in VEEV-induced inflammation and immune response in the brain. In mice infected with the neurovirulent V3000 strain of VEEV, breakdown of the BBB is evident at later stages of infection when the VEEV antigen is localized around inflamed microvessels. The endothelium of the inflamed microvasculature expresses pathogen recognition receptors, chemokines and cellular adhesion molecules that probably aide in the infiltration of peripheral immune cells into the brain parenchyma [37,38,102]. Intracellular adhesion molecule -1 (ICAM-1) knockout mice, which anatomically have a less leaky BBB, exhibit reduced severity of the symptoms and mortality after V3000 infection and demonstrate marked reduction in inflammation of the brain microvasculature [38]. Experiments with VEEV replicon particles (VRPs) where structural genes of V3000 in viral genome were replaced with green fluorescence protein and the resulting RNA was packed in a V3000 envelop, showed that infection of olfactory epithelium was enough to open the BBB. Expression of ICAM-1 and MMP-9, were elevated in the olfactory bulb, cortex, and cerebellum after primary VRP inoculation in the olfactory tract of mice. Secondary VRPs, injected intravenously at a time-point matched with the opening of the BBB by intranasally inoculated primary VRPs, then are able to enter the brain through the opened BBB [100]. VRPs are designed to infect, but not produce live virus particles. Opening of the BBB after infection of the olfactory neurons with VRPs suggests that initial infection itself is sufficient to induce molecular changes in the brain olfactory area that result in alteration of BBB, even though there is no cell-to-cell transmission of the VRP genome. Mice treated with MMP-9 inhibitor show decreased leakage of the BBB coinciding with a significant increase in the mean survival time and reduced mortality post VEEV infection [100].

**Figure 5:**
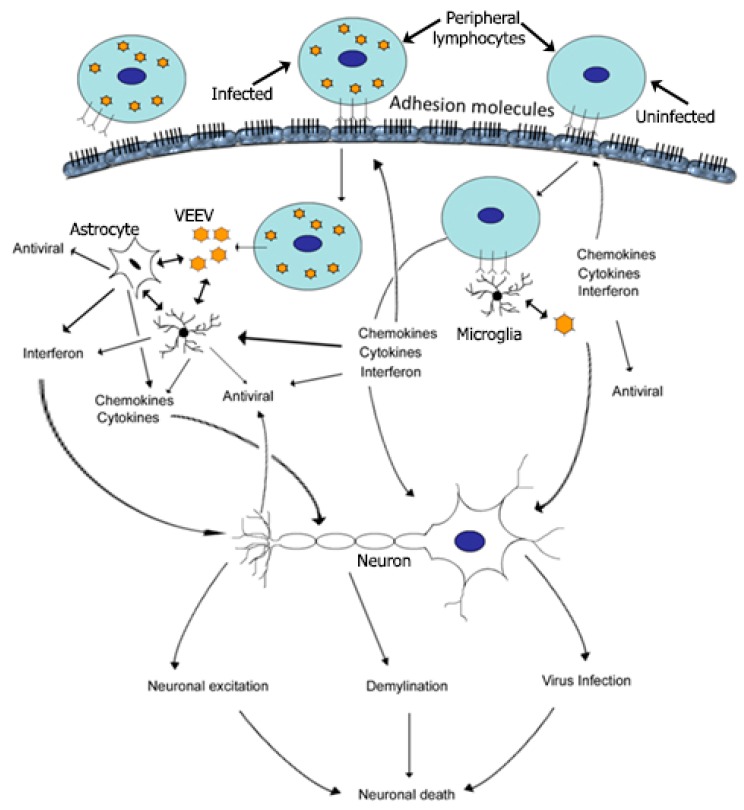
Role of BBB in VEE. Endothelium of the BBB is actively involved in the inflammatory reaction during the CNS phase of VEEV infection. VEEV primarily enters the brain via the olfactory tract replicating first in the olfactory bulb of the brain. Presence of the virus in the brain is detected by the resident glia, the immune cells of the brain. Activation of glial cells release chemokines and cytokines that initiate the first steps of CNS inflammation. The initial inflammatory response probably initiates the alteration in the BBB. Chemokines such as MCP-1 have been shown to be directly involved in opening the BBB. Chemokines and cytokines activate brain microvascular endothelium, resulting in up-regulation of adhesion molecules such as ICAM-1. Ligands of ICAM-1 are expressed on peripheral circulating activated–leucocytes, which may or may not be infected with VEEV. Binding of circulating leucocytes with the brain microvascular endothelium elicits a cascade of events resulting in the transmigration of these cells across the BBB. In the third phase, inflammation and viral load in the brain is augmented by the transmigrating leucocytes. Infected leucocytes increase the viral load in the brain, which further activates the glia resulting in increased inflammatory reactions in the brain. Microglia are antigen presenting cells and may further contribute to an increase in inflammation by presenting the virus to peripheral leucocytes entering the brain and to the resident astrocytes. These events result in a continuous cycle of release of inflammatory cytokines and chemokines in the brain reaching a point where neurons may undergo inflammatory mediated cytotoxic damage in addition to apoptosis induced by direct virus infection.

Secondary invasion of the CNS thus may occur as a combination of virus from low-level infected BBB endothelial cells, peripheral immune cells infected with VEEV, or the free virus circulating in the blood (Figure 5). This secondary invasion is clearly dependent on the primary invasion of the CNS by VEEV via the olfactory tract which contributes to the alterations in the BBB permeability [103]. Mice treated with tunicamycin (TM), a drug that is known to cause BBB damage, exhibited a higher load of VEEV in the brain accompanied by enhanced neuronal death, apoptosis, and inflammation. Tunicamycin treatment increased the titer of virulent V3000 strain of VEEV in the brain of mice, without effecting the serum virus titer. Increased brain titers were associated with manifestation of enhanced encephalitis and faster mortality compared to mice infected with V3000 in absence of TM. Tunicamycin treatment significantly increased the neuroinvasion of V3034, an attenuated VEEV strain, from 11% in untreated mice to 100% in TM-treated mice. In addition, V3034 appeared earlier in the brain tissue of TM-treated mice compared to the untreated mice. Direct inoculation of VEEV in the brain of TM-treated animals did not significantly affect the virus titer levels in the brain compared to intracranial inoculation of VEEV in untreated mice. Since TM alters the BBB barrier, a plausible explanation is that in addition to the entry of VEEV through olfactory tract, the virus also enters the brain through TM-induced damaged BBB, thereby, increasing the viral load and encephalitis in these mice [104]. Interestingly, cadmium (Cd), a heavy metal, has also been shown to increase the titer of V3000 in mouse brains. Although the mechanism of action of Cd increasing brain VEEV titers was not investigated in detail, Cd is known to alter the BBB. It is possible that similar to TM, opening of the BBB by Cd could have increased the viral load in the brain [105,106]. Secondary invasion of the brain by VEEV via the BBB, therefore, is an important aspect of pathogenesis that increases the viral and inflammatory load in the brain and contributes to an adverse outcome of VEEV infection. In the brain, neurons are the primary targets of VEEV. Infection of glia and oligodendrocytes by VEEV appears to follow the neuronal infection [99,107,108]. Viral infection-induced neuronal death is largely via an apoptotic mechanism [107]. Neuronal death may also occur independent of viral infection due to inflammatory cytokine induced cytotoxicity [14,107].

## 6. Inflammation in VEEV Infection

Inflammation is an important aspect of VEEV pathogenesis. In peripheral organs, cellular degeneration is observed during peak viral replication, and tissue recovery is observed with clearing of the virus. For example, in the spleen germinal center activation with mild to extensive necrosis is observed, followed by recovery and restoration of germinal centers [11,16]. In the brain inflammation contributes significantly to the CNS pathology following VEEV infection, and pathological findings include extensive endothelial cuffing, mononuclear cell infiltration, astrogliosis, edema, vacuolation of neuropil, and neuronal karyorrhexis [14,38]. Treatment with anti-inflammatory drugs reduces the initial severity of the symptoms, but it does not protect mice from VEEV-induced mortality, presumably due to direct infection and killing of neuronal cells [38].

Encephalitis in VEEV is associated with the up-regulation of multiple inflammatory mediators such as toll-like receptor signaling, cytokine-inducible nitric oxide synthase (iNOS), tumor necrosis factors alpha (TNF-α), transforming growth factor-beta (TGF-β), interleukins and chemokines [14,37,38,40,109]. Primary astrocytes are sensitive to VEEV infection and release proinflammatory cytokines, TNF-α, and iNOS, in response to virulent V3000 and attenuated V3010 strains of VEEV. VEEV also affects mitochondrial function in addition to the inflammatory response in the brain. Human astrocytoma cells, U87MG, infected with the TC-83 strain of VEEV exhibit decreased mitochondrial membrane potentials and a subsequent increase in the intracellular concentration of reactive oxygen species (ROS). The expression of apoptotic genes and mytophagy was also increased in U87MG cells. In contrast, TC-83 infection of mosquito C6/36cells did not affect the mitochondrial membrane potentials and intracellular ROS levels, although these cells supported TC-83 replication. The study however, is limited by the use of a cancerous cell line that can significantly differ from the primary astrocytes in terms of replication and differential response to VEEV infection [108,110]. Schoneboom and colleagues have shown that very few primary astrocytes undergo apoptosis, and VEEV-induced astrogliosis and inflammation is highly dependent on the virulence of the infecting strain of VEEV. The virulent V3000 strain of VEEV induces a significantly higher inflammatory response and neuronal death compared to the less virulent attenuated strains of VEEV [14]. Cellular adhesion molecules such as ICAM-1 and integrins, and extracellular matrix proteases such as matrix metalloproteases (MMPs), which mediate transvascular migration of lymphocytes into the brain parenchyma, also show increased expression in the brain of VEEV-infected mice [38,111,112,113]. MMPs, in addition to their BBB modulating function, can regulate inflammation either by assisting in the migration of inflammatory cells through the BBB or by direct modification of chemokines and cytokines [114]. Expression of MMPs such as MMP-3, 8, 14, 15 and 16, in addition to chemokines and cytokines such as macrophage chemotactic protein -1 (MCP-1), chemokine (C-X-C motif) ligand (CxCl)-11, TGF-β and interleukin-1 beta (IL-1β), were up-regulated in the brains of VEEV-infected mice. MMP-3 can regulate inflammation by direct cleavage of MCP-1, CxCl-11, and IL-1β [37,38,81,115]. MMPs cleave CxCl-11 to produce a neurotoxin and can activate native forms of TNFα and IL-1β into the active forms [116,117,118]. In contrast, MMP mediated cleavage of MCP-1, TGF-β and IL-1β can result in the inactive forms of these chemokines, suppressing the chemotactic signal for leucocytes and associated inflammation [115,118,119]. Therefore, in addition to the modulation of BBB, MMPs may play a regulatory role in balancing inflammatory responses in VEEV-induced encephalitis. Studies in various knockout mice have highlighted the adverse role of the inflammatory response in VEEV infection. Mice that lack a functional ICAM-1 have a less leaky BBB and exhibit marked reduction in encephalitis following infection with V3000. Low grade encephalitis in ICAM-1 -/- mice is associated with down-regulation of proinflammatory cytokines and differential regulation of anti-inflammatory genes in the brain tissue compared to infection in the wild-type mice. Hind limb paralysis, a characteristic symptom of VEEV infection, was absent in ICAM-1 -/- mice, and 20% survival was observed as compared to the complete mortality in wild-type mice [38]. The role of immune cell-mediated neuroinflammation in VEEV infection is evident in VEEV-infected severe combined immune deficient (SCID) mice. SCID mice have severe combined immunodeficiency spontaneous mutation PRKD^scid^ and lack functional B, T, and NK-cells. Instead of encephalitis, VEEV infection in SCID mice causes spongiosis and leptomeningitis associated with a longer than average survival time and lack of hind limb paralysis [120]. Average survival time after infection with V3000 is also increased in iNOS and TNF receptor knockout mice [14]. Inflammation, therefore, plays a paradoxical role in VEEV infection, either too little or too much of which results in adverse outcomes of the disease. A balanced inflammatory response that can carry out the antiviral response but does not cause autoimmune brain tissue damage will need to be achieved for successful treatment of virulent VEEV infection.

## 7. Vaccines

There is currently no US Food and Drug Administration (FDA)-approved licensed vaccine against VEEV. Several candidate vaccines against VEEV are at various stages of development. These vaccines can be categorized as live-attenuated virus, inactivated virus, recombinant subunit or chimeric virus, virus-like particles, or as passive immunization.

### 7.1. Live-Attenuated Vaccine Candidates

Live-attenuated vaccines are VEEV strains that have been mutated either by sequential passage in cell culture, or by inducing a mutation in the viral genome. The first live-attenuated strain of VEEV that was used for vaccination in humans was TC-83, which was generated by 83 passages of the TrD strain of VEEV in guinea-pig heart cells [121]. TC-83 differs from the parent TrD strain by 11 point mutations resulting in one less nucleotide (11,443nt vs 11,444nt TrD). Attenuation of TC-83 is attributed to two of these mutations, namely, G to A mutation at nucleotide position number 3 in the 5’-noncoding region, and C to G mutation at nucleotide position 8,922 in *E2* gene resulting in Thr to Arg mutation at amino acid position 120 in E2 glycoprotein [21,122]. The TC-83 strain of VEEV is an investigational new drug (IND), No. 142, of the FDA, and is used to vaccinate personnel “at risk” of exposure to VEEV [123]. Live TC-83 is used as a vaccine for immunizing equines in Mexico and Columbia, but is not available in the USA [124]. Currently a multi-center, open label, phase-2 clinical trial is in progress which aims to evaluate the safety and immunogenicity of TC-83 in healthy adults (http://clinicaltrials.gov/ct2/show/study/NCT00582504). TC-83 has several disadvantages that makes it highly unlikely to be approved for mass immunization in humans. TC-83 is reactogenic, and 23-37% of vaccinees report self-contained flu-like symptoms including malaise, headache, fever, chills, nausea, diarrhea, rash, and myalgia [123,125]. TC-83 has a significant environmental risk of dissemination into the wild after immunization, as it was isolated from mosquitoes after the equine immunization drive in the southern states of the USA [126]. TC-83 has a poor responder rate which is suggested to be dependent on the HLA type of the host. Among responders, the antibody titer declines in the first year after immunization requiring booster immunizations for maintenance of protective antibody titer. There is also a potential for reversion to the virulent type [123,127].

Several mutant strains of VEEV have been generated by site directed mutagenesis, which shows differential replication and/or tissue tropism in mice compared to the parent full-length V3000 clone [15,18,19]. One of these live-attenuated strains of VEEV, V3526, was tested as a potential vaccine in clinical trials. V3526 was developed based on the sequence of clonal isolates (J9-1a and J9-1b) of the mutant strain V3022. V3526 was generated by a site directed mutagenesis deleting furin-like cleavage site in E2 precursor (PE2) protein of V3022. The furin cleavage site is needed for final maturation and integration of the E2 glycoprotein in virions. Deletion of this site is lethal for VEEV; however, a second mutation on E1 253 replacing Phe with Ser rescued the virus, resulting in a clone that contains PE2 and E1 in the mature VEEV particle [23,128]. V3526 showed slow replication kinetics in vertebrate as well as non-vertebrate mosquito cells, and was found to be highly attenuated in mice [23]. V3526 demonstrated an excellent safety profile in animal models and provided immunity against both homo- and heterologous VEEV subtypes. In addition, it has minimal environmental hazard due to its reduced transmission potential by mosquitoes compared to TC-83 [128,129,130,131]. However, in phase I clinical trials, V3526 showed adverse reactions including myalgia, lymphopenia, pyrexia, and tachycardia in the vaccinees. Nasal and throat cultures were positive for V3526 and coincided with febrile reactions. Further development of V3526 as a live vaccine candidate was subsequently stopped (http://clinicaltrials.gov/show/NCT00109304) [132]. This observation may have resulted from several reasons: during initial development of the V3526 strain, fast growing large plaque variants were observed which could be an indication that there was a tendency to either revert or mutate into potentially more virulent strains. Additionally, the intracranial inoculation of mice with V3526 showed morbidity and mortality suggesting associated virulence with the strain [23,133]. Since humans are one of the natural hosts of VEEV, the residual virulence of V3526 may have exhibited enhanced levels of adverse events in vaccinees. Nevertheless, V3526 provides a platform that could be used for developing a VEEV vaccine that can provide excellent immunogenicity and heterologous protection against other serotypes of VEEV.

### 7.2. Inactivated Vaccine Candidates

Formalin inactivated TrD strain of VEEV was one of the first inactivated VEEV vaccines to be developed [134]. It was widely used to immunize equines in endemic areas, but residual virulence due to escaped live virus particles presented a significant risk. Indeed, incompletely inactivated vaccines were suspected to be the source of outbreaks of VEEV before 1970 in South, Central, and North America [135,136]. Formalin inactivated TC-83, called C-84, is currently used as an IND for immunizing non-responders to the live TC-83 vaccine and in vaccinees whose antibody titer falls below 1:20 following primary immunization with live TC-83 [123,137]. Formalin inactivated TC-83 is available in combination with eastern and western equine encephalitis virus for immunization of horses in the USA [http://www.merck-animal-health-usa.com]. Formalin inactivated V3526 has also shown protection against an infectious VEEV challenge, but has not formally been included in immunization regimens for equines [138,139].

Another approach for chemical inactivation of VEEV was by 1, 5 Iodonaphthyl azide (INA). INA is a hydrophobic photoactive compound that in association with short exposure to long wavelength ultraviolet (UV) rays demonstrated complete inactivation of V3000, a full-length infectious clone of the wild-type TrD strain of VEEV. INA-inactivated V3000 protected mice from a footpad challenge with infectious V3000 [133,140]. INA is proposed to inactivate by targeting the functionality of the viral envelop proteins; however, treatment of VEEV with INA in the presence of UV rays also abolished the infectivity of the positive-sense RNA genome of VEEV. Experiments using encephalomyocarditis virus (EMCV), a positive-sense RNA virus, suggested that INA may inactivate the viral RNA genome by direct binding [133,141]. INA-inactivation of V3526 was also carried out and intramuscular immunization of mice with INA-inactivated V3526 conferred complete protection against an aerosol challenge with TrD [142].

Inactivation by ionizing gamma radiation has also been used to generate an inactivated VEEV vaccine. For complete inactivation, V3526 was irradiated with a 50kGy dose of gamma radiation (50kGy) resulting in 30–50% loss in epitope integrity. Although gamma-irradiated V3526 completely protected mice against a subcutaneous challenge, protection against an aerosol challenge with virulent TrD was poor, and only 40% survival was observed after the challenge [139,143]. In a separate study, a novel manganese-decapeptide-inorganic phosphate (MDP) complex derived from radiation-resistant bacteria *Deinococcus radiadurans*, could significantly protect epitopes of V3526 from degradation during inactivation with high doses of gamma radiation. Unlike the study by Fine and group [139], a complete inactivation, was observed at 20kGy or higher doses of gamma radiation. While VEEV epitopes were protected by the MDP complex, the viral genome was completely degraded by the high dose gamma radiation. Immunization with V3526 inactivated by gamma radiation in the presence of MDP complex protected 90% of the mice from an aerosol challenge with TrD [144,145]. Doses used for immunization in these studies varied from 0.2 µg to 5 µg, increasing the protective effect from 40% to 90%, respectively. A detailed dose-sparing study will be needed to identify the optimal immunization dose of gamma radiation inactivated V3526 that can provide complete protection against wild-type VEEV. This approach brings new enthusiasm in efforts to develop a safe inactivated VEEV vaccine, as chemical inactivation of VEEV has been plagued with problems of escape live virus particles and poor immunogenicity. Gamma radiation is regularly used in sterilization of laboratory and biological material. High doses of gamma radiation can ensure a reliable, complete, and homogenous inactivation of large batches of virus particles. Therefore, this approach may fare better for FDA approval than other approaches that are currently being tested as VEEV vaccine candidates.

### 7.3. Chimeric Vaccine Candidates

Chimera is defined by the United States Department of Agriculture as “a new hybrid microorganism created by joining nucleic acid fragments from two or more different microorganisms in which each of at least two of the fragments contain essential genes necessary for replication” [146]. VEEV chimeras have been developed to reduce the infectivity of the parent strains for use as vaccines. TC-83 is the lone IND vaccine for VEEV with demonstrable residual virulence. To overcome the residual virulence, structural genes of TC-83 were placed under the regulation of an internal ribosome entry site (IRES) of EMCV. The EMCV-IRES sequence was cloned in the subgenomic (SG) RNA, replacing the 5’UTR of the SG RNA of TC-83. The resulting TC-83 chimera (VEEV/mutSG/IRES) evolved into a large plaque variant in serial passages, which contains an A to G mutation at nt2758 changing Tyr to Cys at codon 370 (VEEV/mutSG/IRES/1). VEEV/mutSG/IRES/1 failed to replicate in live mosquitoes and mosquito cell cultures, and replicated less efficiently in vertebrate cells, and caused reduced mortality in neonatal mice compared to the parent TC-83 strain. Immunization with VEEV/mutSG/IRES/1 provided 80% protection against virulent VEEV subtype IC strain 3908 [147]. In another study, Guerbois and colleagues reinstated the functional SG promoter of TC-83 and placed the capsid gene alone under EMCV-IRES regulation cloning it downstream of the E1 gene. The resulting virus (VEEV/IRES/C) showed better replication potential than VEEV/mutSG/IRES, but was still attenuated compared to parent TC-83 strain. VEEV/IRES/C provided better protection than VEEV/mutSG/IRES against lethal VEEV challenge with 3098 strain of VEEV subtype IC in both infant and adult CD-1 mice [148]. Rossi and colleagues used a similar approach of EMCV-IRES insertion to mutate VEEV subtype IE strain 68U201 in the two sequences described above i.e., 68U201 virus structural protein under EMCV-IRES control (68U201/IRESv1), and capsid alone under EMCV-IRES control (68U201/IRESv2). The 68U2001/IRES showed lack of replication in mosquito cells, while inducing a lower viremia and complete protection in mice against a lethal challenge with wild-type 68U201. 68U201/IRESv1 also protected macaques against aerosol exposure with live 68U201 [149].

The VEEV capsid protein has a nuclear localization sequence that it uses to interact with the host cell nuclear import (importin-α/β) and export (exportin-1) proteins. Mutations in the NLS sequence of the capsid disrupt its interaction with the host cell nuclear import and export proteins. Combining these two approaches, Atasheva and colleagues generated a mutant TC-83 where the capsid nuclear localization sequence was mutated and the capsid gene under EMCV-IRES regulation was cloned downstream of E1 gene (VEEV/IRES-Cm). VEEV/IRES-Cm chimera, as with other VEEV/EMCV-IRES chimeras, was attenuated compared to the parent TC-83 virus and protected mice against lethal challenge with 3908 strain of VEEV [150]. VEEV chimera based on a SINV backbone has also been explored as an attenuated vaccine candidate. The SINV-VEEV chimera was constructed by replacing SINV structural genes with TC-83 genes with final chimera (SIN-83) consisting of 5’UTR, nsp1-4 and 3’UTR of SINV, and structural proteins of TC-83. One passage of SIN-83 in mammalian cells resulted in a point mutation changing Ser to Thr at codon 795 in nsp2 making the chimera virus stable. Stable SIN-83 chimera induced neutralizing antibody response in infant mice and protected against challenge with wild-type VEEV subtype ID and IC [151]. Similar chimeras were constructed using the structural genes of TrD (SIN/TrD), or VEEV subtype ID strain ZPC738 (SIN/ZPC). The resulting SINV/VEEV chimeric viruses were attenuated and protected mice and hamsters from an infectious challenge with ZPC738. Protection conferred by SINV/TC-83 was found to be inferior to the protection conferred by the SINV/TrD or SINV/ZPC chimera, suggesting inferiority of TC-83 immunogenicity as compared to the infectious parent strains of VEEV. However, wild-type virus was able to invade the CNS of vaccinated mice when the challenge was performed via intranasal route, irrespective of the vaccine chimera expressing wild-type or attenuated VEEV structural genes, suggesting suboptimal mucosal immunity induced by SINV/VEEV chimera [151,152].

Chimera expressing TC-83 structural genes using Eilat virus (EILV) has been described. Eliat virus is an alphavirus that replicates in insect cells; however, its replication is restricted in mammalian cells [153]. Structural genes of EILV (C-E3-E2-6k-E1) were replaced with those of the TC-83 strain. The resulting EILV/TC83 chimera retained its restriction in the insect cells replication profile and did not show virulence in neonatal mice. Immunization of mice with ELIV/TC83 provided protection against a virulent VEEV strain 3908 (subtype IC) challenge. Seroconversion rates and neutralizing antibody titers were better than the parent TC-83 strain, but both viruses showed complete protection against wild-type VEEV challenge. Immunization with a trivalent vaccine containing EILV chimera for EEEV and chikungunya virus in addition to TC-83 did not show complete protection (90% animals survived) against challenge with wild-type VEEV [154]. Restricted replication of EILV chimeras is a major advantage eliminating possibilities of virus replication-induced adverse events in vaccinees. The environmental hazard of EILV/TC83 vaccine is expected to be minimal as the chimera is not expected to replicate in vertebrate hosts. Although the presence of the chimera virus in the serum of vaccinated mice needs to be ruled out to support this assumption. EILV/TC83 provided protection against wild-type VEEV as early as 1 day post immunization, which is similar to the protection observed after inoculation with live-attenuated V3032 strain of VEEV. This nonspecific protection is presumably mediated by innate immune response especially IFN-α/β [154,155].

Another chimeric vaccine candidate was generated by cloning TC-83 strain structural gene sequence *E3-E1* in the equine herpes virus type 1 (EHV-1) vector under human cytomegalovirus promoter regulation. EHV-1, an alphaherpesvirus, is a common pathogen of equines that infects a variety of cells including those of human origin. Therefore EHV-1 is an efficient vector backbone for developing chimeric viruses. Furthermore, human serum with antibodies to other alphaviruses does not neutralize EHV-1, reducing the chance of neutralization of chimera by preexisting alphaherpesvirus antibodies in humans. EHV-1/VEEV chimera (rH_VEEV) completely protected mice from a challenge with a high dose of infectious VEEV; however, no neutralizing antibodies against VEEV were detected in the serum of vaccinated mice even though these immunized mice seroconverted to express anti-VEEV IgG and IgG1 [156]. Modified vaccinia virus Ankara (MVA) expressing the structural proteins (E3-E2-6k-E1) of the TrD strain of VEEV has also been tested as a vaccine candidate. The VEEV envelop proteins were cloned in MVA-Bavarian Nordic (MVA-BN) vector under the control of a synthetic PrHyb promoter [157]. MVA-BN vector is replication-deficient due to blockage of the virus assembly and therefore allows the synthesis of the cloned viral proteins while no progeny viruses are produced [158]. Single dose of MVA-BN-VEEV chimera did not seroconvert mice. However, neutralizing antibody titers were observed two weeks after the booster dose and all animals survived the challenge with virulent TrD. MVA-BN-VEEV chimera administered either as a mixture with two other MVA-BN chimera expressing western equine encephalitis virus (WEEV) and EEEV structural proteins, or as a single chimera expressing structural proteins of all the three alphaviruses, did not induce a neutralizing antibody response to VEEV. However, significant protection was observed against challenge with TrD [157]. Neutralizing antibodies have long been considered the gold standard for testing the protective efficacy of vaccines against VEEV; however, low titers of neutralizing antibodies not correlating with the extent of protection by a vaccine candidate have been reported with inactivated VEEV vaccine candidates [142,145]. Non-neutralizing antibodies to the amino terminus of E2 glycoproteins or antibodies to E1 glycoproteins of VEEV has also been shown to protect against challenge with the wild-type virus. Additionally, cell-mediated immunity in the form of cytotoxic T-cells can be protective against VEEV [159,160,161]. It is possible that replication of rH_VEEV triggers protective antibodies against non-neutralizing epitopes of VEEV glycoproteins and/or T-cell activation that mediates protection against challenge with virulent ZPC798. A limitation of rh_VEEV chimera is expression of EHV-1 gM protein, which can potentially induce immunity against the vector itself, thereby, limiting its use to only one kind of vaccine. In addition, preexisting alphaherpesvirus immunity in equines may neutralize the chimera and limit the use of rH_VEEV vaccine in equines [162,163].

### 7.4. Subunit Vaccine Candidate

DNA constructs expressing the structural proteins of VEEV has been used as vaccine candidates. Structural genes (C-E3-E2-6K-E1) of TrD strain of VEEV cloned in mammalian expression vector pWRG7077 and administered via the epidermis using a gene-gun protected mice and macaques against an aerosol challenge with wild-type VEEV. However, a low neutralizing antibody response was observed, and one macaque exhibited viremia post challenge suggesting non-sterile immunity [164,165]. Removal of the capsid from the above structural gene construct cloned in pWRG7077, combined with codon optimization, significantly improved the neutralizing antibody titers even when immunized with lower dose of DNA vaccine. In addition, delivery of the structural gene construct via intramuscular electroporation, protected against an aerosol challenge with infectious VEEV in various animal models [166]. In a phase I clinical trial, DNA vaccine pWRG/VEE, expressing E3-E2-6K-E1 of VEEV, was well tolerated with no adverse events reported in the vaccinees. Intramuscular route of administration showed 100% seroconversion in the recipients, whereas in the intradermal administration group, seroconversion ranged from 62.5–87.5% [167]. Further modification of this DNA vaccine was done to develop a T-cell epitope-based vaccine. Only the sequences of the structural proteins of VEEV that will selectively bind to major histocompatibility complex (MHC) class II molecules during presentation by antigen presenting cells to CD4+ T-cell were included in the antigen design [168]. VEEV envelope protein antigens were expressed along with Ebola virus antigens as a single gene sequence cloned in pWRG7077 expression plasmid. Balb/C and HLA-DR3 (lacking MHC class II molecules) mice immunized intramuscularly with this DNA construct demonstrated dose-dependent cellular immune response; however, neutralizing antibody response and protection provided by this multi-epitope DNA vaccine against TrD in HLD-DR3 mice was modest at best as compared to the immunization with the whole antigen [168]. It is possible that selecting only the HLA binding domain on the virus antigen could have limited the protective epitope population in the vaccine. Additionally, expression of Ebola virus antigens may have interfered with anti-VEEV immune response. DNA plasmids expressing recombinant E2 (containing E2 gene sequences from VEEV IA/B, IE, western and eastern equine encephalitis viruses, and Mucambo virus) and parental (VEEV IA/B) E1 glycoprotein sequences or vice versa induced a cross reactive antibody response to all five viruses after intradermal inoculation. These constructs elicited a strong total anti-VEEV antibody response and protected mice against an aerosol challenge with the infectious TrD strain of VEEV. The expression of recombinant *E2* gene compared to the parental E2 glycoprotein gene was found to be high and corresponded with higher total and neutralizing anti-E2 antibodies in immunized animals [169].

To improve the immunogenicity of the VEEV-DNA vaccine, an infectious clone containing full-length sequence of TC-83 under the control of a CMV promoter in pcDNA3.1-derived plasmid vector was developed. This infectious-DNA (iDNA) vaccine initiated the TC-83 virus replication and elicited an anti-VEEV antibody response in mice after intravenous inoculation. However, the vaccinated mice only showed partial protection against a challenge with virulent VEEV, as 50% of the mice exhibited low-level viremia [170]. Although expression of TC-83 via iDNA approach was stable and demonstrated feasibility of this approach, iDNA-TC-83 vaccine may inherit the problems associated with the safety of the live-attenuated TC-83 strain of VEEV i.e., potential to revert to infectious type and environmental hazard of transmission by mosquitoes. The risk of reversion and non-sterile immunity may preclude further development of the iDNA-TC-83 platform as VEEV vaccine candidate. A different approach to vaccine development was taken by Rico et al. by taking E1 glycoproteins as the antigen for vaccine development [160]. E1 glycoprotein of alphaviruses is responsible for the fusion of viral envelop membrane with that of the endolysosomes, and subsequent release of viral RNA into the host cell cytoplasm. Neutralizing antibodies to VEEV are predominantly against E2 glycoprotein as they interfere with the virus attachment to, and uptake by the host cells. In study by Rico et al., purified E1 glycoprotein of TrD strain of VEEV and WEEV were integrated in cationic liposomes to form lipid-antigen-nucleic acid-complexes (LANACs) containing E1 glycoproteins of VEEV and WEEV. In a prime-boost subcutaneous immunization regimen, LANAC vaccine protected mice from lethal VEEV and WEEV challenge providing sterilizing immunity. Passive transfer of serum from LANAC immunized mice into naïve mice also conferred protection against a challenge with lethal infection of VEEV [160]. LANAC vaccine platform, therefore, provides a powerful tool to develop pan-alphavirus vaccines; however, sterilizing immunity may only be provided against homologues viruses.

The DNA vaccine platform expressing structural genes of VEEV is attractive from the safety standpoint as it completely avoids the potential of reversal of candidate vaccine platforms to the virulent type, a cause of adverse event associated with virus replication. However, low seroconversion rates, requirement of multiple doses, and potential of non-sterile immunity (as observed in the macaque study) are undesirable. Codon optimization addresses the poor immunogenicity issue; however, further dose optimization for immunogenic efficacy is needed.

### 7.5. Replicon Particles as Vaccine Candidates

VRPs, which can replicate only for one round due to the lack of structural genes in the progeny virus, have been shown to protect against an infectious VEEV challenge when administered as early as 6h before challenge. VRP were generated using V3000 backbone, mature VRP consisted of genome containing V3000 5’UTR, nonstructural gene 1–4, 14 nucleotide downstream of the 26s mRNA transcription start site, and 43 nucleotide multiple cloning site. The VRP envelop consisted of E2 and E1 glycoproteins derived from V3000 [171,172]. Similar observations were made in mice administered with a live-attenuated strain of VEEV, V3032, 24 h prior to the challenge with the infectious VEEV [155]. The mechanism of this nonspecific protection is not fully understood; however, activation of the innate immune response and induction of the antiviral state presumably by the release of endogenous type I IFN, may mediate the protection [155,171]. VRPs stimulate antigen specific mucosal immunity, and therefore have been suggested for use as adjuvants in the vaccines [172,173]. Replication-deficient human adenovirus type 5 vector-based VEEV vaccines (Rad/VEEV) expressing the E2 glycoprotein VEEV subtype I A/B have also been developed. For this, structural genes (E3-E2-6K) sequence of TC-83 was cloned in pMV100 plasmids and site directed mutagenesis was used to revert TC-83 E2 glycoprotein to TrD E2 glycoprotein. This modified VEEV structural gene sequence was then cloned into a pMV60 plasmid creating pMV60/VEEV construct. Replication-deficient human adenovirus type 5 (Ad5) containing VEEV structural proteins (Rad/VEEV) was then generated by using homologous recombination between plasmids pMV60/VEEV and pJM17 (containing entire genome of Ad5) in 293 cells. Intranasal immunization with Rad/VEEV protected mice from low-to-intermediate doses of infectious VEEV, but failed to protect against high doses of infectious VEEV [174]. Use of Rad/VEEV as booster following immunization with DNA vaccine encoding the TC-83 E2 glycoprotein significantly increased the protective efficacy of the DNA or Rad/VEEV vaccine against an aerosol challenge from infectious VEEV [175]. Codon optimization, which improved the efficacy of Rad/VEEV expression in mammalian cells, increased levels of VEEV glycoproteins expression compared to non-optimized Rad/VEEV constructs. Anti-VEEV IgG levels were also increased 10-fold in mice immunized with codon-optimized Rad/VEEV over that of immunization with non-optimized Rad/VEEV constructs, subsequently translating into better protection against a challenge with aerosolized infectious VEEV [176]. VRP containing the furin cleavage site mutated PE2 glycoprotein from V3014 strain of VEEV have also been developed and shown to protect mice and non-human primates against VEEV challenge. Protection was also observed when this replicon particle was used in combination with replicon particles expressing eastern equine encephalitis and western equine encephalitis viruses PE2 gene, suggesting potential of a trivalent vaccine against these three alphaviruses [177]. Replication-deficient VRP are an attractive option for developing VEEV vaccine as they eliminate the adverse events associated with live virus replication and the possibility of reversion to the virulent phenotype. However, generation of VRP vaccines based on a VEEV backbone would require a BSL3 facility which is a major limitation for large scale vaccine development. Use of adenovirus or SINV backbone to generate VRP could circumvent the requirement of BSL3 facility, but the protection provided by these replicon particles have moderate efficacy at best.

### 7.6. Passive Immunization

Passive immunization with anti-VEEV antibodies protect against an infectious VEEV challenge (Table 2). Monoclonal antibodies (Mab) against the E2 and E3 glycoproteins of VEEV protect mice from challenge with infectious VEEV; however, antibodies against E1 glycoprotein provide only weak protection against the infectious virus challenge [178,179]. O’Brien and colleagues identified a broadly reactive monoclonal antibody, CUF37-2a, from animals that were first immunized with TC-83 followed by exposure to 6 different serotypes of VEEV (subtypes I, II, III, IV, V, and VI). CUF37-2a was found to be specific to E2 glycoprotein of VEEV and identified all the VEEV subtypes, except the subtype VI, with which it showed a weaker reactivity. In addition, the antibody protected mice from subcutaneous exposure to the wild-type TrD strain of VEEV [180]. Some of these neutralizing monoclonal antibodies have been humanized and tested for efficacy in pre-clinical animal models for potential clinical application [181,182,183,184]. The safety of these humanized antibodies, however, is needed to be tested in humans. Neutralizing humanized monoclonal antibodies can fill the gap left by non-availability of licensed VEEV vaccine for emergency usages in an outbreak. Rigorous protective efficacy studies in various animal models of VEEV disease can help in gaining FDA approval for emergency clinical application under medical countermeasure for potential bioterror threat. Passive immunization with engineered mesenchymal stromal cells has been described. Human umbilical cord perivascular cells were engineered to express a humanized anti-VEEV antibody hu1A4A1. 1A4A1 is a monoclonal antibody that binds to the E2 protein of VEEV. Mice that received a single intramuscular shot of these cells produced a protective anti-VEEV antibody titer and about ten percent of these mice showed persistent antibody titer three months after the administration of engineered cells [185]. However, large scale production of the engineered mesenchymal cells expressing anti-VEEV antibody may be limited by the factors such as aging and potential of mesenchymal stem cells to support malignant transformations [186,187].

A small number of studies have demonstrated that in addition to antibodies, T-cells may protect from VEEV infection. B-cell deficient µMT mice infected with V3533, a mutant strain of VEEV, develop a severe form of the disease, but eventually recover from the infection presumable by T-cell-mediated virus control [161]. Mice that are deficient in α/β T-cells do not respond to VEEV vaccines and are susceptible to lethal VEEV infection [191]. However, upon reconstitution with VEEV specific CD3+ and CD4+ T-cells from VEEV-vaccinated wild-type mice, these mice exhibit protection from a lethal VEEV challenge suggesting protective role of T-cells in VEEV infection [191,192,193].

## 8. Conclusions

In the last 20 years our understanding of VEEV infection, its clinical manifestation, and the underlying mechanisms of viral replication has considerably increased. The focus on VEEV triggered by its potential as a bio-warfare agent has heightened interest in this virus. The RNA genome of VEEV is a positive-sense single-strand assembly of genes necessary for replication and assembly of virions. Viral proteins interact with host proteins to initiate infection, replication, and control of host translation machinery. Efficacy of such an interaction probably determines the virulence of one strain over the other. Studies with various mutant strains highlight the efficacy of the VEEV replication system to undergo adaptive point mutations to overcome deficiencies in replication, and thus evolve into replication efficient phenotypes. Several host factors such as HS, IKK-β, FXRs, DDX-1, and -3 interact with viral proteins and play an important role in VEEV infection and replication. However, there exists a gap in our understanding of host factors that are absolutely required for virus replication and the conditions which drive the adaptive mutations in the virus resulting in stable infectious phenotypes. Evolutionary pressure, such as overall charge on viral proteins or conformational changes that enable better interaction of viral proteins with the host proteins, may be at play in directing the adaptive mutations. A more thorough understanding of such host-virus protein interactions can lead to discovery of novel therapeutic targets which can then lead to anti-VEEV drug development. It is well established that inflammation is an integral part of the anti-VEEV response, and that excessive inflammation in the brain augments disease pathology. While current therapeutic approaches are centered on supportive treatment of disease symptoms, therapies addressing excessive inflammation to control VEEV-induced encephalitis, may prove to be more effective.

Several studies indicate that envelop glycoproteins of the parental wild-type strains of VEEV elicit the most efficient protective immune response against VEEV. Live-attenuated strains derived from the parental strain, chimeric vaccine, or replicons either have residual virulence and/or provide a less than desirable protection against an infectious VEEV challenge. Therefore, an ideal VEEV vaccine candidate will be one that combines the antigenic supremacy of the parental strain envelope glycoproteins, and the safety of a completely inactivated virus. Recent advancement towards achieving these two properties, either by inactivation with gamma radiation in the presence of a protein-protective MDP complex, or chimeric/replicon vaccine candidates with minimal virulence risks, are exciting and provide a promising potential for developing safe and effective VEEV vaccines with FDA approval. Furin cleavage mutant PE2 glycoprotein of VEEV is highly immunogenic and provides heterologous protection against other subtypes of VEEV. Expression of VEEV structural proteins under EMCV-IRES significantly attenuates the virulence of VEEV. A chimera that expresses the VEEV PE2 protein under EMCV-IRES therefore, may have antigenic superiority as well as the required attenuation to induce no adverse event in vaccinees. Live virus vaccines, either attenuated or chimeras, in limited studies have shown to provide protection as early as one day post immunization which can be useful in the event of an outbreak. However, based on the prior experiences with the live-attenuated alphavirus vaccine candidates, it may be difficult to obtain FDA approval for a live-attenuated VEEV vaccine. Developing a multivalent vaccine for prophylaxis against the three alphaviruses i.e., VEEV, EEEV, and WEEV, is most desired; however, antigenic hindrance and poor vaccine candidates for any one of these three viruses can compromise the entire effort. Given the progress made in developing promising VEEV vaccine candidates, a monovalent vaccine against VEEV alone may prove fruitful, especially for use in case VEEV is used as a bio-warfare or bioterror agent.

## Figures and Tables

**Table 1:** Description of mutations in Venezuelan equine encephalitis virus (VEEV) strains and resulting phenotypic changes.

VEEV Strains	Mutation	Phenotype	Reference
TrD	Parent strain (E1 clone)	Virulent wild-type	Kinney et al. [21]
V3000	Full-length clone of TrD Envelope protein (E) 2 170 silent mutation removing *Sma*I E2 239 (Asn to Ile)	Virulent	Grieder et al. [15]
TC-83	5’ noncoding nt 3 G to A nsp3 codon 260 (Ser to Thr) E2 codon 7 (Lys to Asn) E2 codon 85 (His to Tyr) E2 codon 120 (Thr to Arg) E2 codon 192 (Val to Asp) E2 codon 278 (none) E2 codon 296 (Thr to Ile) E1 codon 161 (Leu to Ile) E1 codon 211 (none) 3’ noncoding nt 11,405 UU to U	Attenuated (IND vaccine)	Kinney et al. [21]
V3010	E2 codon 76 (Glu to Lys)	Attenuated	Davis et al. [18]
V3034	E1 codon 272 (Ala to Thr)	Attenuated	Johnston and Smith [22] Davis et al. [18] Grieder et al. [15]
V3032	E2 codon 209 (Glu to Lys)	Attenuated	Johnston and Smith [22] Davis et al. [18] Grieder et al. [15]
V3014	E2 codon 209 (Glu to Lys) E1 codon 272 (Ala to Thr) E2 codon 239 (Ile to Asn)	Attenuated	Davis et al. [18] Grieder et al. [15]
V3533	E2 codon 76 (Glu to Lys) E2 codon 116 (Lys to Glu)	Attenuated	Aronson et al. [15]
V3526	E3 Δ (56-59) (Furin site cleavage)E1 codon 253 (Phe to Ser)	Attenuated	Davis et al. [23]

**Table 2:** VEEV vaccine candidates.

Vaccine Type	Strain/Antigen	Immunity	Status	Reference
Live-attenuated	TC-83	Sterile	IND	Alevizato et al. [123] Pittman et al. [123]
	V3526	Sterile	Phase I	Pratt et al. [128] Holley et al. [132]
Inactivated	Formalin inactivated TrD	Sterile	Equine vaccine (discontinued)	Cole et al. [134]
	Formalin inactivated C84 (TC-83) *	Poor immunogenicity	IND (Booster) Veterinary vaccine	Edelman et al. [137]
	INA-inactivated V3000 and V3526	Sterile	Pre-clinical	Sharma et al. [133] Gupta et al. [142]
	Gamma-irradiated V3526	Sterile	Pre-clinical	Fine et al. [139] Gayen et al. [145]
Chimera	VEEV/mutSG/IRES/1 (TC-83)	Sterile	Pre-clinical	Volkova et al. [147]
	VEEV/IRES/C (TC-83)	Sterile	Pre-clinical	Guerbois et al. [148]
	VEEV/IRESv1 (68U201) VEEV/IRESv2 (68U201)	Sterile	Pre-clinical	Rossi et al. [149]
	VEEV/IRES-Cm (TC-83)	Sterile	Pre-clinical	Atasheva et al. [150]
	SINV/VEEV (TC-83)	Non-sterile	Pre-clinical	Paessler et al. [151]
	SINV/VEEV (TrD)	Non-sterile	Pre-clinical	Paessler et al. [152]
	SINV/VEEV (ZPC738)	Non-sterile	Pre-clinical	Paessler et al. [152]
	EILV/VEEV (TC-83)	Sterile	Pre-clinical	Erasmus et al. [154]
	EHV-1/VEEV (TC-83)	Sterile	Pre-clinical	Rosas et al. [156]
	MVA-BN/VEEV (TrD)	Sterile	Pre-clinical	Hu et al. [157]
Subunit	pWRG7077/VEEV (TrD structural genes)	Non-sterile	Pre-clinical	Riemenschneider et al. [164] Dupuy et al. [165]
	pWRG7077/VEEV (TrD envelope genes)	Sterile	Phase I	Dupuy et al. [166] Hannaman et al. [167]
	pWRG7077/VEEV (TrD Structural genes with T-cell epitope optimized)	Sterile	Pre-clinical	Bounds et al. [168]
	pWRG7077/VEEV (TrD and IE E2)	Sterile	Pre-clinical	Dupuy et al. [169]
	pcDNA3.1/VEEV (TC-83)	Non-sterile	Pre-clinical	Tretyakova et al. [170]
	LANAC (TrD E1)	Sterile	Pre-clinical	Rico et al. [160]
Replicon particles	VEEV VRP (V3000 E2 and E1)	Non-sterile (6h before challenge)	Pre-clinical	Konopka et al. [171]
	Rad/VEEV (TrD E2)	Sterile	Pre-clinical	Phillpotts et al. [174] Williams et al. [176]
	Rad/VEEV (TC-83 E2)	Sterile	Pre-clinical (Booster)	Perkins et al. [175]
	V3014 VRP (V3014 PE2	Sterile	Pre-clinical	Reed et al. [177]
Passive	1A4A-1 Hu1A4A1IgG1-2A	Sterile (Prophylactic) Non-sterile (Therapeutic)	Pre-clinical	Hu et al. [188] Hu et al. [182] Phillpotts et al. [178]
	Hu Mab F5nIgG	Non-sterile	Pre-clinical	Hunt et al. [183]
	Hu1A3B7 (E2)	post infection	Pre-clinical	Goodchild et al. [189] O’Brien et al. [190]
	13D4 (Anti-E3)	Non-sterile	Pre-clinical	Parker et al. [179]
	CUF37-2a (Anti-E2)	Non-sterile	Pre-clinical	O’Brien et al. [180]
	3B4C-4, Hu Mab Hy4-26C	Non-sterile	Pre-clinical	Hunt et al. [184]

* The strain or antigen presented or targeted.

## References

[1] Aguilar P.V., Estrada-Franco J.G., Navarro-Lopez R., Ferro C., Haddow A.D., Weaver S.C. (2011). Endemic venezuelan equine encephalitis in the americas: Hidden under the dengue umbrella. Future Virol..

[2] Garoff H., Hewson R., Opstelten D.J. (1998). Virus maturation by budding. Microbiol. Mol. Biol. Rev..

[3] Alan B., Weaver S.C. (2012). Arboviruses: Alphaviruses, flaviviruses and bunyaviruses: Encephalitis; yellow fever; dengue; haemorrhagic fever; miscellaneous tropical fevers; undifferentiated fever. Medical Microbiology.

[4] Weaver S.C., Ferro C., Barrera R., Boshell J., Navarro J.C. (2004). Venezuelan equine encephalitis. Annu. Rev. Entomol..

[5] Chosewood L.C., Wilson D.E. (2009). Eastern equine encephalitis (EEE) virus, venezuelan equine encephalitis (VEE) virus, and western equine encephalitis (WEE) virus. Biosafety in Microbiological and Biomedical Laboratories.

[6] Watts D.M., Callahan J., Rossi C., Oberste M.S., Roehrig J.T., Wooster M.T., Smith J.F., Cropp C.B., Gentrau E.M., Karabatsos N. (1998). Venezuelan equine encephalitis febrile cases among humans in the peruvian amazon river region. Am. J. Trop. Med. Hyg..

[7] Rivas F., Diaz L.A., Cardenas V.M., Daza E., Bruzon L., Alcala A., De la Hoz O., Caceres F.M., Aristizabal G., Martinez J.W. (1997). Epidemic venezuelan equine encephalitis in la guajira, colombia, 1995. J. Infect. Dis..

[8] Vilcarromero S., Aguilar P.V., Halsey E.S., Laguna-Torres V.A., Razuri H., Perez J., Valderrama Y., Gotuzzo E., Suarez L., Cespedes M. (2010). Venezuelan equine encephalitis and 2 human deaths, Peru. Emerg. Infect. Dis..

[9] Ehrenkranz N.J., Ventura A.K. (1974). Venezuelan equine encephalitis virus infection in man. Annu. Rev. Med..

[10] Zacks M.A., Paessler S. (2010). Encephalitic alphaviruses. Vet. Microbiol..

[11] Jackson A.C., SenGupta S.K., Smith J.F. (1991). Pathogenesis of venezuelan equine encephalitis virus infection in mice and hamsters. Vet. Pathol..

[12] Charles P.C., Walters E., Margolis F., Johnston R.E. (1995). Mechanism of neuroinvasion of venezuelan equine encephalitis virus in the mouse. Virology.

[13] Vogel P., Abplanalp D., Kell W., Ibrahim M.S., Downs M.B., Pratt W.D., Davis K.J. (1996). Venezuelan equine encephalitis in balb/c mice: Kinetic analysis of central nervous system infection following aerosol or subcutaneous inoculation. Arch. Pathol. Lab. Med..

[14] Schoneboom B.A., Catlin K.M., Marty A.M., Grieder F.B. (2000). Inflammation is a component of neurodegeneration in response to venezuelan equine encephalitis virus infection in mice. J. Neuroimmunol..

[15] Grieder F.B., Davis N.L., Aronson J.F., Charles P.C., Sellon D.C., Suzuki K., Johnston R.E. (1995). Specific restrictions in the progression of venezuelan equine encephalitis virus-induced disease resulting from single amino acid changes in the glycoproteins. Virology.

[16] Gupta P., Sharma A., Han J., Yang A., Bhomia M., Knollmann-Ritschel B., Puri R.K., Maheshwari R.K. (2017). Differential host gene responses from infection with neurovirulent and partially-neurovirulent strains of venezuelan equine encephalitis virus. BMC Infect. Dis..

[17] De la Monte S., Castro F., Bonilla N.J., Gaskin de Urdaneta A., Hutchins G.M. (1985). The systemic pathology of venezuelan equine encephalitis virus infection in humans. Am. J. Trop. Med. Hyg..

[18] Davis N.L., Powell N., Greenwald G.F., Willis L.V., Johnson B.J., Smith J.F., Johnston R.E. (1991). Attenuating mutations in the E2 glycoprotein gene of venezuelan equine encephalitis virus: Construction of single and multiple mutants in a full-length cdna clone. Virology.

[19] Davis N.L., Willis L.V., Smith J.F., Johnston R.E. (1989). In vitro synthesis of infectious venezuelan equine encephalitis virus RNA from a cDNA clone: Analysis of a viable deletion mutant. Virology.

[20] Aronson J.F., Grieder F.B., Davis N.L., Charles P.C., Knott T., Brown K., Johnston R.E. (2000). A single-site mutant and revertants arising in vivo define early steps in the pathogenesis of venezuelan equine encephalitis virus. Virology.

[21] Kinney R.M., Johnson B.J., Welch J.B., Tsuchiya K.R., Trent D.W. (1989). The full-length nucleotide sequences of the virulent trinidad donkey strain of venezuelan equine encephalitis virus and its attenuated vaccine derivative, strain TC-83. Virology.

[22] Johnston R.E., Smith J.F. (1988). Selection for accelerated penetration in cell culture coselects for attenuated mutants of venezuelan equine encephalitis virus. Virology.

[23] Davis N.L., Brown K.W., Greenwald G.F., Zajac A.J., Zacny V.L., Smith J.F., Johnston R.E. (1995). Attenuated mutants of venezuelan equine encephalitis virus containing lethal mutations in the PE2 cleavage signal combined with a second-site suppressor mutation in E1. Virology.

[24] Brandstadter J.D., Yang Y. (2011). Natural killer cell responses to viral infection. J. Innate Immun..

[25] Huprikar J., Dal Canto M.C., Rabinowitz S.G. (1990). Protection against lethal venezuelan equine encephalitis (VEE) virus infection by cell-free supernatant obtained from immune spleen cells. J. Neurol. Sci..

[26] Pinto A.J., Morahan P.S., Brinton M.A. (1988). Comparative study of various immunomodulators for macrophage and natural killer cell activation and antiviral efficacy against exotic RNA viruses. Int. J. Immunopharmacol..

[27] Saikh K.U., Lee J.S., Kissner T.L., Dyas B., Ulrich R.G. (2003). Toll-like receptor and cytokine expression patterns of cd56+ t cells are similar to natural killer cells in response to infection with venezuelan equine encephalitis virus replicons. J. Infect. Dis..

[28] Taylor K., Kolokoltsova O., Patterson M., Poussard A., Smith J., Estes D.M., Paessler S. (2012). Natural killer cell mediated pathogenesis determines outcome of central nervous system infection with venezuelan equine encephalitis virus in C3H/HeN mice. Vaccine.

[29] Julander J.G., Skirpstunas R., Siddharthan V., Shafer K., Hoopes J.D., Smee D.F., Morrey J.D. (2008). C3H/HeN mouse model for the evaluation of antiviral agents for the treatment of venezuelan equine encephalitis virus infection. Antiviral Res..

[30] Steele K.E., Davis K.J., Stephan K., Kell W., Vogel P., Hart M.K. (1998). Comparative neurovirulence and tissue tropism of wild-type and attenuated strains of venezuelan equine encephalitis virus administered by aerosol in c3h/hen and balb/c mice. Vet. Pathol..

[31] Gregoire C., Chasson L., Luci C., Tomasello E., Geissmann F., Vivier E., Walzer T. (2007). The trafficking of natural killer cells. Immunol. Rev..

[32] Hart M.K., Pratt W., Panelo F., Tammariello R., Dertzbaugh M. (1997). Venezuelan equine encephalitis virus vaccines induce mucosal iga responses and protection from airborne infection in BALB/c, but not C3H/HeN mice. Vaccine.

[33] Ricklin D., Hajishengallis G., Yang K., Lambris J.D. (2010). Complement: A key system for immune surveillance and homeostasis. Nat. Immunol..

[34] Dunkelberger J.R., Song W.C. (2010). Complement and its role in innate and adaptive immune responses. Cell Res..

[35] Stoermer K.A., Morrison T.E. (2011). Complement and viral pathogenesis. Virology.

[36] Brooke C.B., Schafer A., Matsushima G.K., White L.J., Johnston R.E. (2012). Early activation of the host complement system is required to restrict central nervous system invasion and limit neuropathology during venezuelan equine encephalitis virus infection. J. Gen. Virol..

[37] Sharma A., Maheshwari R.K. (2009). Oligonucleotide array analysis of toll-like receptors and associated signalling genes in venezuelan equine encephalitis virus-infected mouse brain. J. Gen. Virol..

[38] Sharma A., Bhomia M., Honnold S.P., Maheshwari R.K. (2011). Role of adhesion molecules and inflammation in venezuelan equine encephalitis virus infected mouse brain. Virol. J..

[39] Valerol N., Bonilla E., Espina L.M., Maldonado M., Montero E., Anez F., Levy A., Bermudez J., Melean E., Nery A. (2008). Increase of interleukin-1 beta, gamma interferon and tumor necrosis factor alpha in serum and brain of mice infected with the venezuelan equine encephalitis virus. Investig. Clin..

[40] Sharma A., Bhattacharya B., Puri R.K., Maheshwari R.K. (2008). Venezuelan equine encephalitis virus infection causes modulation of inflammatory and immune response genes in mouse brain. BMC Genom..

[41] Koterski J., Twenhafel N., Porter A., Reed D.S., Martino-Catt S., Sobral B., Crasta O., Downey T., DaSilva L. (2007). Gene expression profiling of nonhuman primates exposed to aerosolized venezuelan equine encephalitis virus. FEMS Immunol. Med. Microbiol..

[42] White L.J., Wang J.G., Davis N.L., Johnston R.E. (2001). Role of alpha/beta interferon in venezuelan equine encephalitis virus pathogenesis: Effect of an attenuating mutation in the 5’ untranslated region. J. Virol..

[43] Grieder F.B., Vogel S.N. (1999). Role of interferon and interferon regulatory factors in early protection against venezuelan equine encephalitis virus infection. Virology.

[44] Simmons J.D., White L.J., Morrison T.E., Montgomery S.A., Whitmore A.C., Johnston R.E., Heise M.T. (2009). Venezuelan equine encephalitis virus disrupts stat1 signaling by distinct mechanisms independent of host shutoff. J. Virol..

[45] Yin J., Gardner C.L., Burke C.W., Ryman K.D., Klimstra W.B. (2009). Similarities and differences in antagonism of neuron alpha/beta interferon responses by venezuelan equine encephalitis and sindbis alphaviruses. J. Virol..

[46] Hefti E., Bishop D.H., Dubin D.T., Stollar V. (1975). 5’ nucleotide sequence of sindbis viral RNA. J. Virol..

[47] Strauss J.H., Strauss E.G. (1994). The alphaviruses: Gene expression, replication, and evolution. Microbiol. Rev..

[48] Frolov I., Akhrymuk M., Akhrymuk I., Atasheva S., Frolova E.I. (2012). Early events in alphavirus replication determine the outcome of infection. J. Virol..

[49] Garmashova N., Atasheva S., Kang W., Weaver S.C., Frolova E., Frolov I. (2007). Analysis of venezuelan equine encephalitis virus capsid protein function in the inhibition of cellular transcription. J. Virol..

[50] Garmashova N., Gorchakov R., Frolova E., Frolov I. (2006). Sindbis virus nonstructural protein NSP2 is cytotoxic and inhibits cellular transcription. J. Virol..

[51] Garmashova N., Gorchakov R., Volkova E., Paessler S., Frolova E., Frolov I. (2007). The old world and new world alphaviruses use different virus-specific proteins for induction of transcriptional shutoff. J. Virol..

[52] Atasheva S., Fish A., Fornerod M., Frolova E.I. (2010). Venezuelan equine encephalitis virus capsid protein forms a tetrameric complex with crm1 and importin alpha/beta that obstructs nuclear pore complex function. J. Virol..

[53] Atasheva S., Garmashova N., Frolov I., Frolova E. (2008). Venezuelan equine encephalitis virus capsid protein inhibits nuclear import in mammalian but not in mosquito cells. J. Virol..

[54] Lundberg L., Pinkham C., Baer A., Amaya M., Narayanan A., Wagstaff K.M., Jans D.A., Kehn-Hall K. (2013). Nuclear import and export inhibitors alter capsid protein distribution in mammalian cells and reduce venezuelan equine encephalitis virus replication. Antiviral Res..

[55] Atasheva S., Krendelchtchikova V., Liopo A., Frolova E., Frolov I. (2010). Interplay of acute and persistent infections caused by venezuelan equine encephalitis virus encoding mutated capsid protein. J. Virol..

[56] Bhalla N., Sun C., Metthew Lam L.K., Gardner C.L., Ryman K.D., Klimstra W.B. (2016). Host translation shutoff mediated by non-structural protein 2 is a critical factor in the antiviral state resistance of venezuelan equine encephalitis virus. Virology.

[57] Snyder J.E., Kulcsar K.A., Schultz K.L., Riley C.P., Neary J.T., Marr S., Jose J., Griffin D.E., Kuhn R.J. (2013). Functional characterization of the alphavirus TF protein. J. Virol..

[58] Kendra J.A., de la Fuente C., Brahms A., Woodson C., Bell T.M., Chen B., Khan Y.A., Jacobs J.L., Kehn-Hall K., Dinman J.D. (2017). Ablation of programmed −1 ribosomal frameshifting in venezuelan equine encephalitis virus results in attenuated neuropathogenicity. J. Virol..

[59] Stapleford K.A., Miller D.J. (2010). Role of cellular lipids in positive-sense rna virus replication complex assembly and function. Viruses.

[60] Volkova E., Gorchakov R., Frolov I. (2006). The efficient packaging of venezuelan equine encephalitis virus-specific RNAs into viral particles is determined by nsP1-3 synthesis. Virology.

[61] Michel G., Petrakova O., Atasheva S., Frolov I. (2007). Adaptation of venezuelan equine encephalitis virus lacking 51-nt conserved sequence element to replication in mammalian and mosquito cells. Virology.

[62] Petrakova O., Volkova E., Gorchakov R., Paessler S., Kinney R.M., Frolov I. (2005). Noncytopathic replication of venezuelan equine encephalitis virus and eastern equine encephalitis virus replicons in mammalian cells. J. Virol..

[63] Li C., Guillen J., Rabah N., Blanjoie A., Debart F., Vasseur J.J., Canard B., Decroly E., Coutard B. (2015). Mrna capping by venezuelan equine encephalitis virus nsp1: Functional characterization and implications for antiviral research. J. Virol..

[64] Hardy W.R., Strauss J.H. (1989). Processing the nonstructural polyproteins of sindbis virus: Nonstructural proteinase is in the c-terminal half of NSP2 and functions both in cis and in trans. J. Virol..

[65] Merits A., Vasiljeva L., Ahola T., Kaariainen L., Auvinen P. (2001). Proteolytic processing of semliki forest virus-specific non-structural polyprotein by NSP2 protease. J. Gen. Virol..

[66] Mayuri, Geders T.W., Smith J.L., Kuhn R.J. (2008). Role for conserved residues of sindbis virus nonstructural protein 2 methyltransferase-like domain in regulation of minus-strand synthesis and development of cytopathic infection. J. Virol..

[67] Montgomery S.A., Johnston R.E. (2007). Nuclear import and export of venezuelan equine encephalitis virus nonstructural protein 2. J. Virol..

[68] Kim D.Y., Atasheva S., Frolova E.I., Frolov I. (2013). Venezuelan equine encephalitis virus NSP2 protein regulates packaging of the viral genome into infectious virions. J. Virol..

[69] Foy N.J., Akhrymuk M., Shustov A.V., Frolova E.I., Frolov I. (2013). Hypervariable domain of nonstructural protein nsp3 of venezuelan equine encephalitis virus determines cell-specific mode of virus replication. J. Virol..

[70] Foy N.J., Akhrymuk M., Akhrymuk I., Atasheva S., Bopda-Waffo A., Frolov I., Frolova E.I. (2013). Hypervariable domains of NSP3 proteins of new world and old world alphaviruses mediate formation of distinct, virus-specific protein complexes. J. Virol..

[71] Rupp J.C., Jundt N., Hardy R.W. (2011). Requirement for the amino-terminal domain of sindbis virus NSP4 during virus infection. J. Virol..

[72] Fata C.L., Sawicki S.G., Sawicki D.L. (2002). Alphavirus minus-strand rna synthesis: Identification of a role for Arg183 of the NSP4 polymerase. J. Virol..

[73] Takkinen K., Peranen J., Keranen S., Soderlund H., Kaariainen L. (1990). The semliki-forest-virus-specific nonstructural protein NSP4 is an autoproteinase. Eur. J. Biochem..

[74] Tomar S., Hardy R.W., Smith J.L., Kuhn R.J. (2006). Catalytic core of alphavirus nonstructural protein NSP4 possesses terminal adenylyltransferase activity. J. Virol..

[75] Malygin A.A., Bondarenko E.I., Ivanisenko V.A., Protopopova E.V., Karpova G.G., Loktev V.B. (2009). C-terminal fragment of human laminin-binding protein contains a receptor domain for venezuelan equine encephalitis and tick-borne encephalitis viruses. Biochemistry (Mosc).

[76] Ludwig G.V., Kondig J.P., Smith J.F. (1996). A putative receptor for venezuelan equine encephalitis virus from mosquito cells. J. Virol..

[77] Kolokoltsov A.A., Fleming E.H., Davey R.A. (2006). Venezuelan equine encephalitis virus entry mechanism requires late endosome formation and resists cell membrane cholesterol depletion. Virology.

[78] Bernard K.A., Klimstra W.B., Johnston R.E. (2000). Mutations in the E2 glycoprotein of venezuelan equine encephalitis virus confer heparan sulfate interaction, low morbidity, and rapid clearance from blood of mice. Virology.

[79] Gardner C.L., Hritz J., Sun C., Vanlandingham D.L., Song T.Y., Ghedin E., Higgs S., Klimstra W.B., Ryman K.D. (2014). Deliberate attenuation of chikungunya virus by adaptation to heparan sulfate-dependent infectivity: A model for rational arboviral vaccine design. PLoS Negl. Trop. Dis..

[80] Gardner C.L., Choi-Nurvitadhi J., Sun C., Bayer A., Hritz J., Ryman K.D., Klimstra W.B. (2013). Natural variation in the heparan sulfate binding domain of the eastern equine encephalitis virus e2 glycoprotein alters interactions with cell surfaces and virulence in mice. J. Virol..

[81] Chelladurai P., Seeger W., Pullamsetti S.S. (2012). Matrix metalloproteinases and their inhibitors in pulmonary hypertension. Eur. Respir. J..

[82] Ryman K.D., Gardner C.L., Burke C.W., Meier K.C., Thompson J.M., Klimstra W.B. (2007). Heparan sulfate binding can contribute to the neurovirulence of neuroadapted and nonneuroadapted sindbis viruses. J. Virol..

[83] Israel A. (2010). The IKK complex, a central regulator of NF-kappaB activation. Cold Spring Harb. Perspect. Biol..

[84] Amaya M., Voss K., Sampey G., Senina S., de la Fuente C., Mueller C., Calvert V., Kehn-Hall K., Carpenter C., Kashanchi F. (2014). The role of ikkbeta in venezuelan equine encephalitis virus infection. PLoS ONE.

[85] Brown V., Jin P., Ceman S., Darnell J.C., O’Donnell W.T., Tenenbaum S.A., Jin X., Feng Y., Wilkinson K.D., Keene J.D. (2001). Microarray identification of FMRP-associated brain mRNAs and altered mRNA translational profiles in fragile X syndrome. Cell.

[86] Kim D.Y., Reynaud J.M., Rasalouskaya A., Akhrymuk I., Mobley J.A., Frolov I., Frolova E.I. (2016). New world and old world alphaviruses have evolved to exploit different components of stress granules, FXR and G3BP proteins, for assembly of viral replication complexes. PLoS Pathog..

[87] Amaya M., Brooks-Faulconer T., Lark T., Keck F., Bailey C., Raman V., Narayanan A. (2016). Venezuelan equine encephalitis virus non-structural protein 3 (nsp3) interacts with RNA helicases DDX1 and DDX3 in infected cells. Antiviral Res..

[88] Atasheva S., Akhrymuk M., Frolova E.I., Frolov I. (2012). New parp gene with an anti-alphavirus function. J. Virol..

[89] Atasheva S., Frolova E.I., Frolov I. (2014). Interferon-stimulated poly(adp-ribose) polymerases are potent inhibitors of cellular translation and virus replication. J. Virol..

[90] Poddar S., Hyde J.L., Gorman M.J., Farzan M., Diamond M.S. (2016). The interferon-stimulated gene IFITM3 restricts infection and pathogenesis of arthritogenic and encephalitic alphaviruses. J. Virol..

[91] Muehlenbein M.P., Cogswell F.B., James M.A., Koterski J., Ludwig G.V. (2006). Testosterone correlates with venezuelan equine encephalitis virus infection in macaques. Virol. J..

[92] Ryzhikov A.B., Ryabchikova E.I., Sergeev A.N., Tkacheva N.V. (1995). Spread of venezuelan equine encephalitis virus in mice olfactory tract. Arch. Virol..

[93] Park C.H., Ishinaka M., Takada A., Kida H., Kimura T., Ochiai K., Umemura T. (2002). The invasion routes of neurovirulent a/hong kong/483/97 (H5N1) influenza virus into the central nervous system after respiratory infection in mice. Arch. Virol..

[94] Harberts E., Yao K., Wohler J.E., Maric D., Ohayon J., Henkin R., Jacobson S. (2011). Human herpesvirus-6 entry into the central nervous system through the olfactory pathway. Proc. Natl. Acad. Sci. USA.

[95] Faber H.K., Silverberg R.J., Dong L. (1944). Poliomyelitis in the cynomolgus monkey: Iii. Infection by inhalation of droplet nuclei and the nasopharyngeal portal of entry, with a note on this mode of infection in rhesus. J. Exp. Med..

[96] Plakhov I.V., Arlund E.E., Aoki C., Reiss C.S. (1995). The earliest events in vesicular stomatitis virus infection of the murine olfactory neuroepithelium and entry of the central nervous system. Virology.

[97] Lafay F., Coulon P., Astic L., Saucier D., Riche D., Holley A., Flamand A. (1991). Spread of the CVS strain of rabies virus and of the avirulent mutant avo1 along the olfactory pathways of the mouse after intranasal inoculation. Virology.

[98] Yamada M., Nakamura K., Yoshii M., Kaku Y., Narita M. (2009). Brain lesions induced by experimental intranasal infection of Japanese encephalitis virus in piglets. J. Comp. Pathol..

[99] Gorelkin L. (1973). Venezuelan equine encephalomyelitis in an adult animal host. An electron microscopic study. Am. J. Pathol..

[100] Schafer A., Brooke C.B., Whitmore A.C., Johnston R.E. (2011). The role of the blood-brain barrier during venezuelan equine encephalitis virus infection. J. Virol..

[101] Steele K.E., Twenhafel N.A. (2010). Review paper: Pathology of animal models of alphavirus encephalitis. Vet. Pathol..

[102] Schafer A., Whitmore A.C., Konopka J.L., Johnston R.E. (2009). Replicon particles of venezuelan equine encephalitis virus as a reductionist murine model for encephalitis. J. Virol..

[103] Cain M.D., Salimi H., Gong Y., Yang L., Hamilton S.L., Heffernan J.R., Hou J., Miller M.J., Klein R.S. (2017). Virus entry and replication in the brain precedes blood-brain barrier disruption during intranasal alphavirus infection. J. Neuroimmunol..

[104] Steele K.E., Seth P., Catlin-Lebaron K.M., Schoneboom B.A., Husain M.M., Grieder F., Maheshwari R.K. (2006). Tunicamycin enhances neuroinvasion and encephalitis in mice infected with venezuelan equine encephalitis virus. Vet. Pathol..

[105] Shukla A., Shukla G.S., Srimal R.C. (1996). Cadmium-induced alterations in blood-brain barrier permeability and its possible correlation with decreased microvessel antioxidant potential in rat. Hum. Exp. Toxicol..

[106] Seth P., Husain M.M., Gupta P., Schoneboom A., Grieder B.F., Mani H., Maheshwari R.K. (2003). Early onset of virus infection and up-regulation of cytokines in mice treated with cadmium and manganese. Biometals.

[107] Jackson A.C., Rossiter J.P. (1997). Apoptotic cell death is an important cause of neuronal injury in experimental venezuelan equine encephalitis virus infection of mice. Acta Neuropathol..

[108] Schoneboom B.A., Fultz M.J., Miller T.H., McKinney L.C., Grieder F.B. (1999). Astrocytes as targets for venezuelan equine encephalitis virus infection. J. Neurovirol..

[109] Schoneboom B.A., Lee J.S., Grieder F.B. (2000). Early expression of IFN-alpha/beta and inos in the brains of venezuelan equine encephalitis virus-infected mice. J. Interferon Cytokine Res..

[110] Keck F., Brooks-Faulconer T., Lark T., Ravishankar P., Bailey C., Salvador-Morales C., Narayanan A. (2017). Altered mitochondrial dynamics as a consequence of venezuelan equine encephalitis virus infection. Virulence.

[111] Frank P.G., Lisanti M.P. (2008). ICAM-1: Role in inflammation and in the regulation of vascular permeability. Am. J. Physiol. Heart Circ. Physiol..

[112] Taddei A., Giampietro C., Conti A., Orsenigo F., Breviario F., Pirazzoli V., Potente M., Daly C., Dimmeler S., Dejana E. (2008). Endothelial adherens junctions control tight junctions by VE-cadherin-mediated upregulation of claudin-5. Nat. Cell Biol..

[113] Ley K., Laudanna C., Cybulsky M.I., Nourshargh S. (2007). Getting to the site of inflammation: The leukocyte adhesion cascade updated. Nat. Rev. Immunol..

[114] Parks W.C., Wilson C.L., Lopez-Boado Y.S. (2004). Matrix metalloproteinases as modulators of inflammation and innate immunity. Nat. Rev. Immunol..

[115] McQuibban G.A., Gong J.H., Wong J.P., Wallace J.L., Clark-Lewis I., Overall C.M. (2002). Matrix metalloproteinase processing of monocyte chemoattractant proteins generates CC chemokine receptor antagonists with anti-inflammatory properties in vivo. Blood.

[116] Zhang K., McQuibban G.A., Silva C., Butler G.S., Johnston J.B., Holden J., Clark-Lewis I., Overall C.M., Power C. (2003). HIV-induced metalloproteinase processing of the chemokine stromal cell derived factor-1 causes neurodegeneration. Nat. Neurosci..

[117] Mohan M.J., Seaton T., Mitchell J., Howe A., Blackburn K., Burkhart W., Moyer M., Patel I., Waitt G.M., Becherer J.D. (2002). The tumor necrosis factor-alpha converting enzyme (tace): A unique metalloproteinase with highly defined substrate selectivity. Biochemistry.

[118] Schonbeck U., Mach F., Libby P. (1998). Generation of biologically active IL-1 beta by matrix metalloproteinases: A novel caspase-1-independent pathway of IL-1 beta processing. J. Immunol..

[119] Maeda S., Dean D.D., Gomez R., Schwartz Z., Boyan B.D. (2002). The first stage of transforming growth factor beta1 activation is release of the large latent complex from the extracellular matrix of growth plate chondrocytes by matrix vesicle stromelysin-1 (mmp-3). Calcif. Tissue Int..

[120] Charles P.C., Trgovcich J., Davis N.L., Johnston R.E. (2001). Immunopathogenesis and immune modulation of venezuelan equine encephalitis virus-induced disease in the mouse. Virology.

[121] Berge T.O., Banks I.S., Tigertt W.D. (1961). Attenuation of venezuelan equine encephalomyelitis virus by in vitro cultivation in guinea-pig heart cells. Am. J. Hyg..

[122] Kinney R.M., Chang G.J., Tsuchiya K.R., Sneider J.M., Roehrig J.T., Woodward T.M., Trent D.W. (1993). Attenuation of venezuelan equine encephalitis virus strain tc-83 is encoded by the 5’-noncoding region and the E2 envelope glycoprotein. J. Virol..

[123] Pittman P.R., Makuch R.S., Mangiafico J.A., Cannon T.L., Gibbs P.H., Peters C.J. (1996). Long-term duration of detectable neutralizing antibodies after administration of live-attenuated vee vaccine and following booster vaccination with inactivated vee vaccine. Vaccine.

[124] Paessler S., Weaver S.C. (2009). Vaccines for venezuelan equine encephalitis. Vaccine.

[125] Alevizatos A.C., McKinney R.W., Feigin R.D. (1967). Live, attenuated venezuelan equine encephalomyelitis virus vaccine. I. Clinical effects in man. Am. J. Trop Med. Hyg..

[126] Pedersen C.E., Robinson D.M., Cole F.E. (1972). Isolation of the vaccine strain of venezuelan equine encephalomyelitis virus from mosquitoes in louisiana. Am. J. Epidemiol..

[127] Erwin-Cohen R., Porter A., Pittman P., Rossi C., Dasilva L. (2012). Host responses to live-attenuated venezuelan equine encephalitis virus (TC-83): Comparison of naive, vaccine responder and nonresponder to TC-83 challenge in human peripheral blood mononuclear cells. Hum. Vaccin. Immunother..

[128] Pratt W.D., Davis N.L., Johnston R.E., Smith J.F. (2003). Genetically engineered, live attenuated vaccines for venezuelan equine encephalitis: Testing in animal models. Vaccine.

[129] Rao V., Hinz M.E., Roberts B.A., Fine D. (2004). Environmental hazard assessment of venezuelan equine encephalitis virus vaccine candidate strain V3526. Vaccine.

[130] Fine D.L., Roberts B.A., Teehee M.L., Terpening S.J., Kelly C.L., Raetz J.L., Baker D.C., Powers A.M., Bowen R.A. (2007). Venezuelan equine encephalitis virus vaccine candidate (V3526) safety, immunogenicity and efficacy in horses. Vaccine.

[131] Reed D.S., Lind C.M., Lackemeyer M.G., Sullivan L.J., Pratt W.D., Parker M.D. (2005). Genetically engineered, live, attenuated vaccines protect nonhuman primates against aerosol challenge with a virulent IE strain of venezuelan equine encephalitis virus. Vaccine.

[132] Holley P., Fine D.L., Terpening S.J., Mallory C.J., Main C.A., Snow D.M. Safety of an attenuated venezuelan equine encephalitis virus (VEEV) vaccine in humans. Proceedings of the 48th ICAAC/IDSA.

[133] Sharma A., Gupta P., Glass P.J., Parker M.D., Maheshwari R.K. (2011). Safety and protective efficacy of ina-inactivated venezuelan equine encephalitis virus: Implication in vaccine development. Vaccine.

[134] Cole F.E., May S.W., Robinson D.M. (1973). Formalin-inactivated venezuelan equine encephalomyelitis (trinidad strain) vaccine produced in rolling-bottle cultures of chicken embryo cells. Appl. Microbiol..

[135] Kinney R.M., Tsuchiya K.R., Sneider J.M., Trent D.W. (1992). Molecular evidence for the origin of the widespread venezuelan equine encephalitis epizootic of 1969 to 1972. J. Gen. Virol..

[136] Weaver S.C., Pfeffer M., Marriott K., Kang W., Kinney R.M. (1999). Genetic evidence for the origins of venezuelan equine encephalitis virus subtype IAB outbreaks. Am. J. Trop Med. Hyg..

[137] Edelman R., Ascher M.S., Oster C.N., Ramsburg H.H., Cole F.E., Eddy G.A. (1979). Evaluation in humans of a new, inactivated vaccine for venezuelan equine encephalitis virus (C-84). J. Infect. Dis..

[138] Martin S.S., Bakken R.R., Lind C.M., Garcia P., Jenkins E., Glass P.J., Parker M.D., Hart M.K., Fine D.L. (2010). Evaluation of formalin inactivated v3526 virus with adjuvant as a next generation vaccine candidate for venezuelan equine encephalitis virus. Vaccine.

[139] Fine D.L., Jenkins E., Martin S.S., Glass P., Parker M.D., Grimm B. (2010). A multisystem approach for development and evaluation of inactivated vaccines for venezuelan equine encephalitis virus (VEEV). J. Virol. Methods.

[140] Sharma A., Raviv Y., Puri A., Viard M., Blumenthal R., Maheshwari R.K. (2007). Complete inactivation of venezuelan equine encephalitis virus by 1,5-iodonaphthylazide. Biochem. Biophys. Res. Commun..

[141] Gupta P., Sharma A., Mathias V., Raviv Y., Blumenthal R., Maheshwari R.K. (2015). Inactivation of non-enveloped virus by 1,5 iodonaphthyl azide. BMC Res. Notes.

[142] Gupta P., Sharma A., Spurgers K.B., Bakken R.R., Eccleston L.T., Cohen J.W., Honnold S.P., Glass P.J., Maheshwari R.K. (2016). 1,5-iodonaphthyl azide-inactivated V3526 protects against aerosol challenge with virulent venezuelan equine encephalitis virus. Vaccine.

[143] Martin S.S., Bakken R.R., Lind C.M., Garcia P., Jenkins E., Glass P.J., Parker M.D., Hart M.K., Fine D.L. (2010). Comparison of the immunological responses and efficacy of gamma-irradiated V3526 vaccine formulations against subcutaneous and aerosol challenge with venezuelan equine encephalitis virus subtype IAB. Vaccine.

[144] Gaidamakova E.K., Myles I.A., McDaniel D.P., Fowler C.J., Valdez P.A., Naik S., Gayen M., Gupta P., Sharma A., Glass P.J. (2012). Preserving immunogenicity of lethally irradiated viral and bacterial vaccine epitopes using a radio- protective Mn^2+^-peptide complex from deinococcus. Cell Host Microbe.

[145] Gayen M., Gupta P., Morazzani E.M., Gaidamakova E.K., Knollmann-Ritschel B., Daly M.J., Glass P.J., Maheshwari R.K. (2017). Deinococcus Mn^2+^-peptide complex: A novel approach to alphavirus vaccine development. Vaccine.

[146] Biologics C.f.V. (2005). Chimera as an additional naming convention for live recombinant products. CENTER FOR VETERINARY BIOLOGICS NOTICE NO. 05-23.

[147] Volkova E., Frolova E., Darwin J.R., Forrester N.L., Weaver S.C., Frolov I. (2008). IRES-dependent replication of venezuelan equine encephalitis virus makes it highly attenuated and incapable of replicating in mosquito cells. Virology.

[148] Guerbois M., Volkova E., Forrester N.L., Rossi S.L., Frolov I., Weaver S.C. (2013). IRES-driven expression of the capsid protein of the venezuelan equine encephalitis virus tc-83 vaccine strain increases its attenuation and safety. PLoS Negl. Trop Dis..

[149] Rossi S.L., Guerbois M., Gorchakov R., Plante K.S., Forrester N.L., Weaver S.C. (2013). Ires-based venezuelan equine encephalitis vaccine candidate elicits protective immunity in mice. Virology.

[150] Atasheva S., Kim D.Y., Frolova E.I., Frolov I. (2014). Venezuelan equine encephalitis virus variants lacking transcription inhibitory functions demonstrate highly attenuated phenotype. J. Virol..

[151] Paessler S., Fayzulin R.Z., Anishchenko M., Greene I.P., Weaver S.C., Frolov I. (2003). Recombinant sindbis/venezuelan equine encephalitis virus is highly attenuated and immunogenic. J. Virol..

[152] Paessler S., Ni H., Petrakova O., Fayzulin R.Z., Yun N., Anishchenko M., Weaver S.C., Frolov I. (2006). Replication and clearance of venezuelan equine encephalitis virus from the brains of animals vaccinated with chimeric SIN/VEE viruses. J. Virol..

[153] Nasar F., Palacios G., Gorchakov R.V., Guzman H., Da Rosa A.P., Savji N., Popov V.L., Sherman M.B., Lipkin W.I., Tesh R.B. (2012). Eilat virus, a unique alphavirus with host range restricted to insects by RNA replication. Proc. Natl. Acad. Sci. USA.

[154] Erasmus J.H., Seymour R.L., Kaelber J.T., Kim D.Y., Leal G., Sherman M.B., Frolov I., Chiu W., Weaver S.C., Nasar F. (2018). Novel insect-specific eilat virus-based chimeric vaccine candidates provide durable, mono- and multivalent, single-dose protection against lethal alphavirus challenge. J. Virol..

[155] Grieder F.B., Davis B.K., Zhou X.D., Chen S.J., Finkelman F.D., Gause W.C. (1997). Kinetics of cytokine expression and regulation of host protection following infection with molecularly cloned venezuelan equine encephalitis virus. Virology.

[156] Rosas C.T., Paessler S., Ni H., Osterrieder N. (2008). Protection of mice by equine herpesvirus type 1 based experimental vaccine against lethal venezuelan equine encephalitis virus infection in the absence of neutralizing antibodies. Am. J. Trop Med. Hyg..

[157] Hu W.G., Steigerwald R., Kalla M., Volkmann A., Noll D., Nagata L.P. (2018). Protective efficacy of monovalent and trivalent recombinant MVA-based vaccines against three encephalitic alphaviruses. Vaccine.

[158] Timm A., Enzinger C., Felder E., Chaplin P. (2006). Genetic stability of recombinant MVA-BN. Vaccine.

[159] Hunt A.R., Short W.A., Johnson A.J., Bolin R.A., Roehrig J.T. (1991). Synthetic peptides of the E2 glycoprotein of venezuelan equine encephalomyelitis virus. II. Antibody to the amino terminus protects animals by limiting viral replication. Virology.

[160] Rico A.B., Phillips A.T., Schountz T., Jarvis D.L., Tjalkens R.B., Powers A.M., Olson K.E. (2016). Venezuelan and western equine encephalitis virus e1 liposome antigen nucleic acid complexes protect mice from lethal challenge with multiple alphaviruses. Virology.

[161] Brooke C.B., Deming D.J., Whitmore A.C., White L.J., Johnston R.E. (2010). T cells facilitate recovery from venezuelan equine encephalitis virus-induced encephalomyelitis in the absence of antibody. J. Virol.

[162] Fausther-Bovendo H., Kobinger G.P. (2014). Pre-existing immunity against ad vectors: Humoral, cellular, and innate response, what’s important?. Hum. Vaccin. Immunother..

[163] Saxena M., Van T.T., Baird F.J., Coloe P.J., Smooker P.M. (2013). Pre-existing immunity against vaccine vectors--friend or foe?. Microbiology.

[164] Riemenschneider J., Garrison A., Geisbert J., Jahrling P., Hevey M., Negley D., Schmaljohn A., Lee J., Hart M.K., Vanderzanden L. (2003). Comparison of individual and combination DNA vaccines for b. Anthracis, ebola virus, marburg virus and venezuelan equine encephalitis virus. Vaccine.

[165] Dupuy L.C., Richards M.J., Reed D.S., Schmaljohn C.S. (2010). Immunogenicity and protective efficacy of a DNA vaccine against venezuelan equine encephalitis virus aerosol challenge in nonhuman primates. Vaccine.

[166] Dupuy L.C., Richards M.J., Ellefsen B., Chau L., Luxembourg A., Hannaman D., Livingston B.D., Schmaljohn C.S. (2011). A DNA vaccine for venezuelan equine encephalitis virus delivered by intramuscular electroporation elicits high levels of neutralizing antibodies in multiple animal models and provides protective immunity to mice and nonhuman primates. Clin. Vaccine Immunol..

[167] Hannaman D., Dupuy L.C., Ellefsen B., Schmaljohn C.S. (2016). A phase 1 clinical trial of a DNA vaccine for venezuelan equine encephalitis delivered by intramuscular or intradermal electroporation. Vaccine.

[168] Bounds C.E., Terry F.E., Moise L., Hannaman D., Martin W.D., De Groot A.S., Suschak J.J., Dupuy L.C., Schmaljohn C.S. (2017). An immunoinformatics-derived DNA vaccine encoding human class II T cell epitopes of ebola virus, sudan virus, and venezuelan equine encephalitis virus is immunogenic in HLA transgenic mice. Hum. Vaccin Immunother..

[169] Dupuy L.C., Locher C.P., Paidhungat M., Richards M.J., Lind C.M., Bakken R., Parker M.D., Whalen R.G., Schmaljohn C.S. (2009). Directed molecular evolution improves the immunogenicity and protective efficacy of a venezuelan equine encephalitis virus DNA vaccine. Vaccine.

[170] Tretyakova I., Lukashevich I.S., Glass P., Wang E., Weaver S., Pushko P. (2013). Novel vaccine against venezuelan equine encephalitis combines advantages of DNA immunization and a live attenuated vaccine. Vaccine.

[171] Konopka J.L., Thompson J.M., Whitmore A.C., Webb D.L., Johnston R.E. (2009). Acute infection with venezuelan equine encephalitis virus replicon particles catalyzes a systemic antiviral state and protects from lethal virus challenge. J. Virol..

[172] Thompson J.M., Whitmore A.C., Konopka J.L., Collier M.L., Richmond E.M., Davis N.L., Staats H.F., Johnston R.E. (2006). Mucosal and systemic adjuvant activity of alphavirus replicon particles. Proc. Natl. Acad. Sci. USA.

[173] Thompson J.M., Nicholson M.G., Whitmore A.C., Zamora M., West A., Iwasaki A., Staats H.F., Johnston R.E. (2008). Nonmucosal alphavirus vaccination stimulates a mucosal inductive environment in the peripheral draining lymph node. J. Immunol..

[174] Phillpotts R.J., O’Brien L., Appleton R.E., Carr S., Bennett A. (2005). Intranasal immunisation with defective adenovirus serotype 5 expressing the venezuelan equine encephalitis virus E2 glycoprotein protects against airborne challenge with virulent virus. Vaccine.

[175] Perkins S.D., O’Brien L.M., Phillpotts R.J. (2006). Boosting with an adenovirus-based vaccine improves protective efficacy against venezuelan equine encephalitis virus following DNA vaccination. Vaccine.

[176] Williams A.J., O’Brien L.M., Phillpotts R.J., Perkins S.D. (2009). Improved efficacy of a gene optimised adenovirus-based vaccine for venezuelan equine encephalitis virus. Virol. J..

[177] Reed D.S., Glass P.J., Bakken R.R., Barth J.F., Lind C.M., da Silva L., Hart M.K., Rayner J., Alterson K., Custer M. (2014). Combined alphavirus replicon particle vaccine induces durable and cross-protective immune responses against equine encephalitis viruses. J. Virol..

[178] Phillpotts R.J. (2006). Venezuelan equine encephalitis virus complex-specific monoclonal antibody provides broad protection, in murine models, against airborne challenge with viruses from serogroups I, II and III. Virus Res..

[179] Parker M.D., Buckley M.J., Melanson V.R., Glass P.J., Norwood D., Hart M.K. (2010). Antibody to the e3 glycoprotein protects mice against lethal venezuelan equine encephalitis virus infection. J. Virol..

[180] O’Brien L.M., Underwood-Fowler C.D., Goodchild S.A., Phelps A.L., Phillpotts R.J. (2009). Development of a novel monoclonal antibody with reactivity to a wide range of venezuelan equine encephalitis virus strains. Virol. J..

[181] Porta J., Jose J., Roehrig J.T., Blair C.D., Kuhn R.J., Rossmann M.G. (2014). Locking and blocking the viral landscape of an alphavirus with neutralizing antibodies. J. Virol..

[182] Hu W.G., Phelps A.L., Jager S., Chau D., Hu C.C., O’Brien L.M., Perkins S.D., Gates A.J., Phillpotts R.J., Nagata L.P. (2010). A recombinant humanized monoclonal antibody completely protects mice against lethal challenge with venezuelan equine encephalitis virus. Vaccine.

[183] Hunt A.R., Bowen R.A., Frederickson S., Maruyama T., Roehrig J.T., Blair C.D. (2011). Treatment of mice with human monoclonal antibody 24h after lethal aerosol challenge with virulent venezuelan equine encephalitis virus prevents disease but not infection. Virology.

[184] Hunt A.R., Frederickson S., Hinkel C., Bowdish K.S., Roehrig J.T. (2006). A humanized murine monoclonal antibody protects mice either before or after challenge with virulent venezuelan equine encephalomyelitis virus. J. Gen. Virol..

[185] Braid L.R., Hu W.G., Davies J.E., Nagata L.P. (2016). Engineered mesenchymal cells improve passive immune protection against lethal venezuelan equine encephalitis virus exposure. Stem Cells Transl. Med..

[186] Kim H.J., Park J.S. (2017). Usage of human mesenchymal stem cells in cell-based therapy: Advantages and disadvantages. Dev. Reprod..

[187] Zhang Q., Xiang W., Yi D.Y., Xue B.Z., Wen W.W., Abdelmaksoud A., Xiong N.X., Jiang X.B., Zhao H.Y., Fu P. (2018). Current status and potential challenges of mesenchymal stem cell-based therapy for malignant gliomas. Stem Cell Res. Ther..

[188] Hu W.G., Chau D., Wu J., Jager S., Nagata L.P. (2007). Humanization and mammalian expression of a murine monoclonal antibody against venezuelan equine encephalitis virus. Vaccine.

[189] Goodchild S.A., O’Brien L.M., Steven J., Muller M.R., Lanning O.J., Logue C.H., D’Elia R.V., Phillpotts R.J., Perkins S.D. (2011). A humanised murine monoclonal antibody with broad serogroup specificity protects mice from challenge with venezuelan equine encephalitis virus. Antiviral Res..

[190] O’Brien L.M., Goodchild S.A., Phillpotts R.J., Perkins S.D. (2012). A humanized murine monoclonal antibody protects mice from venezuelan equine encephalitis virus, everglades virus and mucambo virus when administered up to 48 h after airborne challenge. Virology.

[191] Paessler S., Yun N.E., Judy B.M., Dziuba N., Zacks M.A., Grund A.H., Frolov I., Campbell G.A., Weaver S.C., Estes D.M. (2007). Alpha-beta t cells provide protection against lethal encephalitis in the murine model of VEEV infection. Virology.

[192] Taylor K., Kolokoltsova O., Ronca S.E., Estes M., Paessler S. (2017). Live, attenuated venezuelan equine encephalitis virus vaccine (TC83) causes persistent brain infection in mice with non-functional alphabeta T-cells. Front. Microbiol..

[193] Yun N.E., Peng B.H., Bertke A.S., Borisevich V., Smith J.K., Smith J.N., Poussard A.L., Salazar M., Judy B.M., Zacks M.A. (2009). Cd4+ T cells provide protection against acute lethal encephalitis caused by venezuelan equine encephalitis virus. Vaccine.

